# Long-Term Consequences of COVID-19 in Predominantly Immunonaive Patients: A Canadian Prospective Population-Based Study

**DOI:** 10.3390/jcm12185939

**Published:** 2023-09-13

**Authors:** Justine Benoit-Piau, Karine Tremblay, Alain Piché, Frédéric Dallaire, Mathieu Bélanger, Marc-André d’Entremont, Jean-Charles Pasquier, Martin Fortin, Catherine Bourque, Fanny Lapointe, Jean-François Betala-Belinga, Geneviève Petit, Guillaume Jourdan, Renata Bahous, Camilo Maya, Amira Benzina, Muhammad Faiyaz Hossain, Marie-Audrey Peel, Olivier Houle, Marie-Sandrine Auger, Antoine Rioux, Paul Farand

**Affiliations:** 1 Research Center of the Centre Hospitalier Universitaire de Sherbrooke (CHUS), University of Sherbrooke, Sherbrooke, QC J1H 5N4, Canada; justine.benoit-piau@usherbrooke.ca; 2 Pharmacology and Physiology Department, Faculty of Medicine and Health Sciences, University of Sherbrooke, Sherbrooke, QC J1H 5N4, Canada; karine.tremblay@usherbrooke.ca (K.T.); fanny.lapointe@usherbrooke.ca (F.L.); 3 Department of Microbiology and Infectiology, University of Sherbrooke, Sherbrooke, QC J1H 5N4, Canada; alain.piche@usherbrooke.ca (A.P.); guillaume.jourdan@usherbrooke.ca (G.J.); 4 Department of Pediatrics, University of Sherbrooke, Sherbrooke, QC J1H 5N4, Canada; frederic.a.dallaire@usherbrooke.ca; 5 Department of Family and Emergency Medicine, University of Sherbrooke, Sherbrooke, QC J1H 5N4, Canada; mathieu.belanger@usherbrooke.ca (M.B.); martin.fortin@usherbrooke.ca (M.F.); 6 Division of Cardiology, Department of Medicine, Faculty of Medicine and Health Sciences, University of Sherbrooke, Sherbrooke, QC J1H 5N4, Canada; marc-andre.dentremont@usherbrooke.ca (M.-A.d.); catherine.bourque@usherbrooke.ca (C.B.); 7 Population Health Research Institute, Hamilton, ON L8L 2X2, Canada; 8 Department of Obstetrics and Gynecology, University of Sherbrooke, Sherbrooke, QC J1H 5N4, Canada; jean-charles.pasquier@usherbrooke.ca; 9 Direction of Saguenay-Lac-Saint-Jean Public Health Department, Saguenay, QC J1H 5N4, Canada; jean-francois.betala-belinga@usherbrooke.ca; 10 Department of Community Health Sciences, Faculty of Medicine and Health Sciences, University of Sherbrooke, Sherbrooke, QC J1H 5N4, Canada; genevieve.petit@usherbrooke.ca; 11 Faculty of Medicine and Health Sciences, University of Sherbrooke, Sherbrooke, QC J1H 5N4, Canada; renata.bahous@usherbrooke.ca (R.B.); camilo.maya@usherbrooke.ca (C.M.); amira.benzina@usherbrooke.ca (A.B.); muhammad.faiyaz.hossain@usherbrooke.ca (M.F.H.); marie-audrey.peel@usherbrooke.ca (M.-A.P.); olivier.houle3@usherbrooke.ca (O.H.); marie-sandrine.auger@usherbrooke.ca (M.-S.A.); antoine.rioux3@usherbrooke.ca (A.R.)

**Keywords:** COVID-19, post-acute COVID-19 syndrome, health-related quality of life, risk factors

## Abstract

**Background**: Lingering symptoms are frequently reported after acute SARS-CoV-2 infection, a condition known as post-COVID-19 condition (PCC). The duration and severity of PCC in immunologically naïve persons remain unclear. Furthermore, the long-term consequences of these chronic symptoms on work and mental health are poorly documented. **Objective**: To determine the outcome, the risk factors, and the impact on work and mental health associated with post-COVID-19 symptoms. **Methods**: This prospective population-based study assessed acute COVID-19 symptoms and their evolution for up to nine months following infection. Individuals aged 18 years and older with COVID-19 in three Canadian regions between 1 November 2020 and 31 May 2021 were recruited. Participants completed a questionnaire that was either administered by trained student investigators over the phone or self-administered online. **Results**: A total of 1349 participants with a mean age of 46.6 ± 16.0 years completed the questionnaire. Participants were mostly unvaccinated at the time of their COVID-19 episode (86.9%). Six hundred and twenty-two participants (48.0%) exhibited one symptom or more, at least three months post-COVID-19. Among participants with PCC, 23.0% to 37.8% experienced fatigue at the time of survey. Moreover, 6.1% expressed psychological distress. Risk factors for PCC and fatigue included female sex (OR = 1.996), higher number of symptoms (OR = 1.292), higher severity of episode (OR = 3.831), and having a mental health condition prior to the COVID-19 episode (OR = 5.155). **Conclusions**: In this multicenter cohort study, almost half (47%) of the participants reported persistent symptoms >3 months after acute infection. Baseline risk factors for PCC include female sex, number and severity of symptoms during acute infection, and a previous diagnosis of mental health disorder. Having PCC negatively impacted health-related quality of life and these patients were more likely to exhibit psychological distress, as well as fatigue.

## 1. Introduction

SARS-CoV-2 is a single-stranded RNA virus responsible for the coronavirus disease (COVID-19). COVID-19 has caused over 4.3 million infections and 46 thousand deaths in Canada thus far [1]. The disease was first thought to mainly affect the respiratory system. However, various studies rapidly showed that it affects multiple other systems [2,3,4]. In order to reduce mortality, healthcare resources were at first overwhelmingly focused on acutely ill patients and those exhibiting greater severity in symptoms [5]. Growing evidence suggests that a number of COVID-19 survivors exhibit long-term sequelae, also known as post-COVID-19 conditions (PCC). Common symptoms associated with PCC include fatigue, post-exertional fatigue, cognitive impairments, headaches, insomnia, and cardiopulmonary problems [6]. Due to the heterogenous severity of lingering symptoms, these people do not always seek medical attention [4,5]. Thus, a majority of experts agree that PCC should be managed by an interdisciplinary team [7].

Multiple cohort studies have investigated PCC in various populations [8,9,10]. These studies allowed for identifying common symptoms suffered through PCC and a number of risk factors. However, many of these studies had limitations including small sample size; inclusion of hospitalized individuals only, not limited to polymerase chain reaction (PCR) proven infections; and inclusion of specific groups, such as only healthcare workers. Moreover, few studies investigated the impact of PCC on quality of life. There remain significant gaps in our understanding of PCC, and our healthcare systems are still struggling to accommodate all of the specific needs of these patients [11]. Indeed, care trajectories have yet to be completely established [11].

To help improve our understanding of PCC, we recruited participants with documented SARS-CoV-2 infection, irrespective of disease severity. The primary objective was to document the prevalence of PCC after SARS-CoV-2 infection. Our secondary objectives were (1) to compare characteristics of PCC between three Canadian regions, (2) to compare characteristics of patients with PCC to those without PCC, and (3) to identify risk factors of PCC.

## 2. Methods

### 2.1. Setting and Design

This prospective population-based study was conducted in close collaboration with the Public Health Departments of three administrative regions in Canada: Estrie, Saguenay-Lac-Saint-Jean, and New Brunswick. A stratified block randomization was used to select a sample of individuals with SARS-CoV-2-proven infection from each of the study regions. The random selection was completed in multiple steps. Briefly, all subjects with a positive PCR test between 1 November 2020 and 31 May 2021 were first divided by region and age range. The second step was to determine how many participants per region and age range were needed. A number was assigned to every participant and randomly reorganized. The first participants corresponding to the required number per region and age range were selected. The details of sample size calculation and randomization are provided in Supplementary S1. The questionnaires were either administered by a trained third-year medical student investigator over the phone or self-administered by the participant online. This study was registered with ClinicalTrials (NCT03928509).

### 2.2. Participants and Ethics

Participants were randomly sampled among persons diagnosed with COVID-19 (SARS-CoV-2 PCR positive) residing in one of the study regions between November 2020 and May 2021. The study protocol was approved by the appropriate institutional research ethics boards (MP-31-2019-3172). Public Health Departments of Saguenay-Lac-Saint-Jean and New Brunswick provided a list of patients having consented to be contacted for research purposes. The Public Health Department of Estrie, in its framework of population monitoring, identified people that could be reached for the study purposes. Informed consent was obtained from all study participants prior to study participation.

Eligibility criteria included (1) a COVID-19 diagnosis confirmed using a SARS-CoV-2 PCR test, with or without symptoms; (2) being reachable by phone or e-mail for the duration of the study, and (3) being aged 18 years or older. Participants were excluded if they were deceased at the time of study, if their health would not allow the questionnaire to be completed, if they were unfit, or could not provide informed consent. All participants were at least 12 weeks post-COVID-19.

### 2.3. Data Collection

Data collection was carried out by third-year students of the undergraduate medical program at Université de Sherbrooke. Participants were invited to respond to the study questionnaire over the phone or online, at their convenience. Student investigators were trained and standardized to administer the survey questionnaire by the study principal investigator (PF) and the study coordinator (JBP).

Data were collected during the months of August and September of 2021. All data entry was carried out in REDCap (Research Electronic Data Capture, Vanderbilt University, Nashville, TN, USA) by either student investigators or participants [12].

### 2.4. Questionnaires and Data

Participants’ sociodemographic profiles were established from responses to study questions relating to language preference, year of birth, sex at birth, gender, education level, socioeconomic level, occupation, time off occupation due to confinement, and time off occupation due to COVID-19 episode.

Data collected on COVID-19 included self-reported information on episode severity (asymptomatic, symptomatic, hospitalized), access to a physician or a nurse practitioner, and vaccination status. The list of symptoms investigated was based on a 25-symptom list established by the Quebec COVID-19 Biobank (“Biobanque Québécoise de la COVID-19”) [13]. Fatigue in adult participants was examined using the Fatigue Severity Scale (FSS) and the Schedule of Fatigue and Anergia/General Practice (SOFA/GP) [14,15].

Participants were asked whether they had the following long-term health conditions: cancer, pulmonary diseases, cardiovascular diseases, digestive, endocrine and urinary diseases, musculoskeletal conditions, and mental health conditions. In addition, they were asked about their cardiovascular capacities using selected questions from the Duke Activity Scale Index (M-DASI-4Q) [16].

Participants’ lifestyle information addressed their fruit and vegetable consumption, height, and weight. Participants 18 years and older were also asked about tobacco, alcohol, and cannabis consumption [17].

Participants’ mental health status was evaluated using the 6-item Kessler Psychological Distress Scale [18].

The World Health Organization (WHO) defines PCC as the persistence of at least one symptom of COVID-19 three months after the initial episode, lasting for at least 2 months, and not being better explained by another diagnosis [19]. This definition was used in the current study. Since all participants were at least three months post-COVID-19, symptoms had been lingering for at least 2 months. The questions were designed in such a way that the symptoms were asked in relation to their COVID-19 episode to ensure that they could not be explained by another pathology. The clinical severity of the initial infection was defined according to WHO severity scale [20].

The complete English and French questionnaires administered are available in Supplementary S2.

### 2.5. Data Analysis

All analyses were performed using SPSS 28.0 (Statistical Package for the Social Sciences, IBM, Armonk, NY, USA). Descriptive analyses were used to characterize participants. For exploratory comparisons between regions, chi-squared tests and ANOVAs were used with Bonferroni corrections. A classification tree analysis was also performed to identify what combination of factors best differentiates between individuals with and without PCC.

## 3. Results

### 3.1. Participation Rate

During the study period, 9048 adults ≥ 18 years received a positive PCR COVID-19 result in Estrie. Of these, 4400 were randomly selected and prorated based on relative population age group size. Out of the 5526 individuals diagnosed with COVID-19 in Saguenay-Lac-Saint-Jean, 1200 agreed to be contacted for research purposes. Of these, 400 adults were randomly selected and prorated based on relative population age group size. In New Brunswick, between November 2020 and May 2021, out of 1857 individuals diagnosed with COVID-19, a total of 698 agreed to be contacted for research on COVID-19.

**Figure 1:**
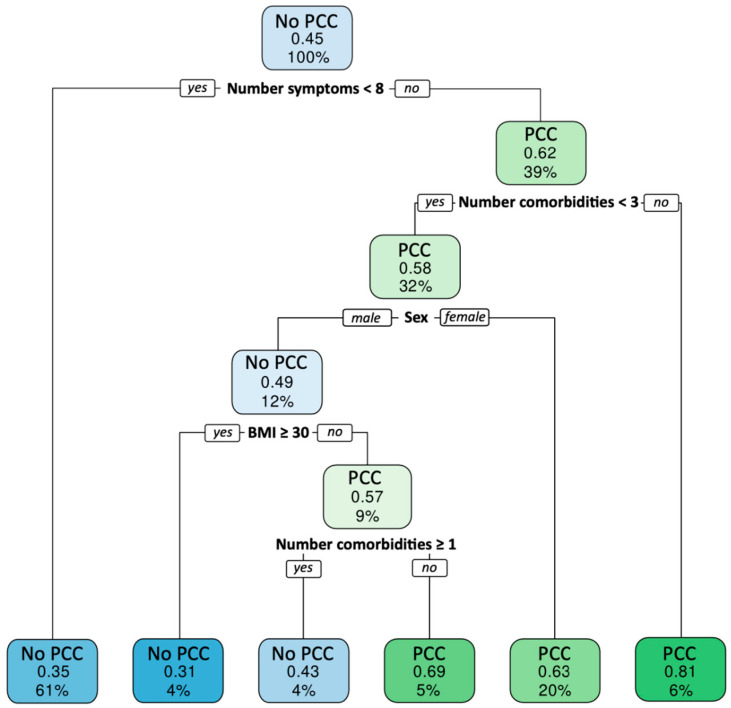
PCC classification tree.

Figure 1 shows the participant flow chart. During the 2021 data collection, out of a total of 6884 selected eligible individuals, we attempted to contact 6741 (97.9%) participants at least once. Among the 4266 individuals reached, a total of 3318 (77.8%) agreed to participate. A total of 592 (17.8%) responded to the survey questionnaire administered over the phone. Of the 2398 who agreed to self-administer the questionnaire online, 969 (40.4%) eventually completed it. Overall, 1349 respondents completed the questionnaire, yielding an overall response rate of 43.8% (1367/3124).

### 3.2. Clinical Characteristics of the Study Cohort

As shown in Table 1, mean age was 46.7 ± 16.1 years old. Most participants were women (58.2%), spoke French (91.9%), and were full-time workers (55.0%). Most participants had a college or university diploma (53.0%). Of 1376 participants, 311 (27.3%) were healthcare workers. Most participants reported eating between two and four portions of fruits or vegetables daily (62.5%), never smoked tobacco (58.2%), never participated in binge drinking (49.0%), and never used recreational cannabis (86.0%).

Most common comorbidities included musculoskeletal conditions (22.4%), mental health conditions (21.9%), and cardiovascular diseases (20.6%) (Table 1). Interestingly, while 67.5% of participants reported having a stable mental health condition prior to their COVID-19 episode, 22.7% reported that their mental health condition had worsened, 4.0% reported that the condition had improved, and 5.8% had a new diagnosis of mental health condition since their COVID-19 episode.

### 3.3. Acute COVID-19 Characteristics

As shown in Table 2, most participants had a mild episode of COVID-19 (159 asymptomatic (12.0%) and 1100 symptomatic (82.8%)). Few had moderate (4.0%) or severe (1.2%) episodes based on WHO severity scale [20]. The most reported symptoms included constitutional (73.6%), neurological (69.0%), and otorhinolaryngology manifestations (64.9%). The majority of participants (86.9%) were immunonaive, that is, unvaccinated against COVID-19, or had received one dose (11.0%), and only 2.1% were considered fully vaccinated (received at least two doses).

Participants showed very few functional disabilities three to nine months after their COVID-19 episode. The majority returned to work within four weeks (87.8%). Other participants returned to work one to three months after their episode (7.2%), more than three months after their episode (2.9%) or did not return to work at all (1.9%). Regarding access to healthcare professionals, 17.0% of participants did not have a practitioner or nurse practitioner. Additionally, 17.3% had difficulties with the appointment system and 17.4% had limited access due to the unavailability of professionals.

### 3.4. Post-COVID-19 Conditions

As shown in Table 3, among participants with PCC (n = 622, 48.0%), symptoms at onset were most often neurological (83.5%) or constitutional (80.9%). The symptoms most often reported at the time of survey 3 to 9 months (median of 6 months) after their COVID-19 episode were neurological (59.3%) and musculoskeletal (43.9%). As for fatigue, there were 31.9% of participants who had a score higher or equal to five for the FSS and 37.8% who had a score higher or equal to two for the SOFA. When combined, 23.0% of participants met both criteria for fatigue. Psychological distress was reported by 6.1% of participants with PCC compared to 1.2% by those without PCC.

As shown in Table 4, participants with PCC were older and had a higher BMI than participants who did not have PCC (*p* ≤ 0.010). Moreover, they were predominantly female (*p* < 0.001). They also showed a higher prevalence of comorbidities (*p* < 0.001) and number of symptoms (*p* < 0.001), and a higher proportion of moderate or severe episode (*p* < 0.001).

Risk factors at baseline regarding the onset of PCC and PCC with fatigue are shown in Table 5. Baseline risk factors for developing PCC included age (OR = 1.010), being a female (OR = 1.383), and a higher BMI (OR = 1.029). All comorbidities were associated with an increased risk of having PCC without fatigue (OR ≥ 1.340). The severity of episode (OR = 1.764) and number of symptoms (OR = 1.136) were also risk factors of PCC. Being a health professional also increased this likelihood (OR = 1.038). Risk factors were similar for PCC with fatigue. Age (OR = 1.012), being a female (OR = 1.996), and higher BMI (OR = 1.056) were risk factors. Again, all comorbidities were linked to a higher risk of PCC (OR ≥ 2.421). The severity of episode (OR = 3.831) and number of symptoms at onset (OR = 1.292) were also risk factors for PCC and fatigue. Being a health professional also put participants at risk of PCC with fatigue (OR = 1.525).

Figure 1 presents the classification tree analysis showing that the number of symptoms at the initial presentation (with a cutoff of 8) is the main discriminant factor to determine who will have PCC or not. Other factors determined by the algorithm, but with less weight, are number of comorbidities, sex, and BMI.

## 4. Discussion

### 4.1. Main Observations

To our knowledge, this study is the largest to date to describe long-term functional impairments of COVID-19 in a Canadian population. Most patients included in our study had a mild episode of COVID-19 and were not hospitalized. This is consistent with reported distribution of symptom severity in the population [8,10]. Another strength of this study is the high number of participants with functional data and extensive characterization of fatigue. Also, all participants included in the study had a PCR test confirming the infection.

The prevalence of PCC in our study population was 48%. That proportion is congruent with other studies [10,21,22,23,24]. Describing functional impacts in patients with PCC is essential since it could affect the burden of their condition. Patients who experience one minor symptom more than 3 months post-infection could be different than patients experiencing PCC who also reported fatigue or other functional impairments. Fatigue was reported by more than 30% of participants with PCC, making it a major manifestation of this condition. Prevalence of post-COVID-19 fatigue has been reported for other countries [25]. This study adds to our understanding of the prevalence of fatigue post-COVID-19 in a Canadian population. We also used a well-known fatigue score to identify the 15.2% of patients with PCC who also experience fatigue.

Among participants with functional impairments, being slower than most people of the same age on flat ground or being short of breath was described by only 7.6% of participants with PCC. Although COVID-19 and PCC can severely affect cardiovascular capacities for some people, the populational burden seems to be mild for that aspect. In a similar way, in our study, very few patients (1.9%) completely ceased their occupation in the long term. It should however be noted that 12.7% of patients who continued an occupation found it more demanding. Among the symptoms reported by patients with PCC at the time of the survey, the most frequently reported were neurological symptoms, including fatigue and cognitive and functional impairments. These symptoms could be linked to other long-term functional impacts of COVID-19 [26,27]. These symptoms could limit functional capacities and aptitude to be active and work.

We also explored factors that can predict the occurrence of PCC. Consistent with other studies, we found that the numbers of symptoms at onset is the main predictor of PCC [22,23]. We also observed that participants with PCC without fatigue showed a higher prevalence of comorbidities and higher number of symptoms at onset. When we look at participants with PCC and fatigue, we see that the odds ratios were even higher for being female and for the presence of comorbidities. Among them, mental health condition was the comorbidity with the higher odds ratio. These patients showed a higher prevalence of comorbidities that could be linked with less efficient coping mechanisms and lesser physical reserves [28].

All lingering symptoms and functional impacts reported in the current study should be interpreted considering the absence of a control group. The negative background effect brought by the pandemic could be a confounder [29]. Indeed, studies have found that psychological impacts were present in the overall population in the wake of the pandemic, not only in people who were infected with COVID-19 [30]. Moreover, the pandemic has had an impact on the workforce of the general population.

With regard to the timeframe of the study, patients included in this cohort were mainly unvaccinated or partially vaccinated related to the prevalence of vaccination at the time of recruitment. The exact proportion of COVID-19 variants among our study population is unknown. However, based on epidemiological data, we know that there was not a predominant variant at the beginning of our study period and that Alpha was the predominant variant at the end of the study period [31]. These epidemiological observations and the low vaccination coverage at the time of the study could explain the high rate of PCC observed. The proportion of patients who will experience PCC and the burden of fatigue as well as functional impacts with different variants and with the increased vaccination coverage is unknown. This will be documented in a follow-up study. This future study will also describe the evolution of patients who suffered from PCC in the 2021 survey.

### 4.2. Limitations

This study has inherent selection and information biases in its design that were considered in the data collection process by the application of preventive measures or in the interpretation of the results that were carefully made.

With regard to the selection bias, the subjects were selected randomly among the COVID-19-positive patients of the public health records, but only those who could be reached by phone participated in this study. This, as well as the timing of call periods, may have biased participants’ characteristics. We explored data about the COVID-19-positive patients of the Estrie region who did not complete the survey. They have the same sex distribution (women 56.4% for patients included in the study vs. 53.7% for patients who did not complete the questionnaire), but less patients who participated in the study were over 70 years old (9.0% of the sample vs. 20.0% of the COVID-19-positive patients). This later finding can be explained by many of the screening tests for patients over 70 years old occurring among patients residing in nursing homes with health conditions, making participation in this kind of study difficult. We do not think that this difference interfered significantly with our results.

In addition, the volunteer bias of the study sampling limits our generalization of the results to the whole population. However, since we did not collect the reasons of refusal to participate or the retrospective data on COVID-19 patients having died from the disease or other causes, we cannot stipulate that our sample is representative of all SARS-CoV-2-infected individuals. Since the primary objective of this study was to compile data on PCC, absence of retrospective data from deceased COVID-19 patients should not bias interpretation of the results.

Another limitation of this study may result from possible information biases. Firstly, a chronological bias in recruitment of the subjects may have introduced a misclassification of the PCC conditions. Indeed, some of the subjects were 3 months post-infection at the time of recruitment while others were at 6 months post-infection. Such delay may underestimate the PCC status (as per the WHO definition) in this sample. Moreover, it should be considered that although questions were designed to identify lingering symptoms from the participants’ COVID-19 episode, it cannot be entirely ruled out that symptoms may be due to other causes. Secondly, the self-reported retrospective data are submitted to the recall bias for which we have no way to verify the veracity (no data collected from medical records). This bias may affect measured frequencies of the data such as symptoms or other health conditions at COVID-19 diagnosis given the time lapse between diagnosis and questionnaire administration. To mitigate this, most questions were formulated to gather information regarding symptoms in the week or month prior to the survey’s administration. Thirdly, although the majority of participants were unvaccinated, some were partially or fully vaccinated. This could have impacted the prevalence of PCC among participants [24,32]. Finally, the large number of student investigator interviewers could be viewed as an observer bias. However, all of those involved in data collection were trained on and standardized to the questionnaire used in this study and trained on how to record information.

## 5. Conclusions

This study describes the long-term functional impacts of the COVID-19 pandemic among a Canadian population. It was found that having comorbidities prior to the COVID-19 episode, particularly mental health conditions, is the main predictor of PCC. Participants with PCC and fatigue were predominantly women and had more symptoms at onset and a higher severity of episode. This research contributes to our understanding of the intricate pathophysiology of PCC.

## Figures and Tables

**Table 1:** Characteristics of COVID-19-positive study participants.

	Total ^a^ N = 1349	Estrie N = 824	SLSJ N = 434	New-Brunswick N = 91	*p*-Value ^b^
Anthropometry and demographics					
Age in years (mean ± SD)	46.6 ± 16.0	46.6 ± 16.5	46.0 ± 15.1	49.5 ± 15.7	0.163
Sex (n of men (%))	522 (41.9)	327 (43.7)	156 (38.0)	39 (44.3)	0.160
BMI in kg/m^2^ (mean ± SD)	27.8 ± 5.6	27.7 ± 5.7	27.7 ± 5.5	28.7 ± 5.8	0.515
Language (n (%))					
French	1239 (91.8)	762 (92.7)	430 (99.1)	47 (51.6)	<0.001
English	78 (5.8)	34 (4.1)	3 (0.7) ^†^	41 (45.1) ^†^	
Others	30 (2.2)	26 (3.2) ^‡^	1 (0.2) ^†^	3 (3.3)	
Environment (n (%))					
Private household	1012 (75.1)	582 (70.6) ^†^	362 (83.6)	68 (74.7)	
Apartment	296 (22.0)	217 (26.3)	60 (13.9) ^†^	19 (20.9)	<0.001
Private residence for seniors or CHSLD	24 (1.8)	17 (2.1)	5 (1.2)	2 (2.2)	
Others	16 (1.2)	8 (1.0)	6 (1.4)	2 (2.2)	
Main occupation (n (%))					
Self-employed	111 (8.3)	69 (8.4)	36 (8.4)	6 (6.7)	
Full-time worker	741 (55.2)	439 (53.5)	253 (58.7)	49 (54.4)	
Part-time worker	82 (6.1)	52 (6.3)	23 (5.3)	7 (7.8)	0.452
Student	98 (7.3)	68 (8.3)	27 (6.3)	3 (3.3)	
Retired	194 (14.5)	125 (15.2)	52 (12.1)	17 (18.9)	
Others	116 (8.6)	68 (8.3)	40 (9.3)	8 (8.9)	
Education (n (%))					
No certificate	91 (7.4)	68 (9.2)	18 (4.4) ^†^	5 (5.7)	
High school diploma	278 (22.5)	196 (26.4)	60 (14.7) ^†^	22 (25.3)	
Apprenticeship or trade	209 (16.9)	115 (15.5)	80 (19.7)	14 (16.1)	
school diploma or other					
College diploma	349 (28.2)	189 (25.4)	142 (34.9) ^†^	18 (20.7)	<0.001
University diploma	310 (25.1)	175 (23.6)	107 (26.3)	28 (32.2)	
Income (n (%))					
<CAD 19,999 to CAD 29,999	151 (13.9)	100 (15.8)	42 (11.2)	9 (12.2)	
CAD 30,000 to CAD 89,999	514 (47.5)	317 (50.0)	162 (43.2)	35 (47.3)	
>CAD 90,000	418 (38.6)	217 (34.2) ^‡^	171 (45.6) ^†^	30 (40.5) ^‡†^	0.007
Health and social services worker (n (%))	308 (27.3)	194 (28.6)	83 (22.3)	31 (39.7)	0.003
Lifestyle (in consumption)					
Fruits and vegetables in portions (n (%))					
Five or more	266 (21.4)	164 (21.9)	82 (20.1)	20 (23.3)	
Between 2 and 4	778 (62.7)	467 (62.4)	259 (63.6)	52 (60.5)	0.948
Less than 2	197 (15.9)	117 (15.6)	66 (16.2)	14 (16.3)	
Tobacco in smoking (n (%))					
Current	152 (12.2)	84 (11.2)	62 (15.0)	6 (6.9)	
Former	370 (29.6)	214 (28.5)	126 (30.5)	30 (34.5)	0.091
Never	739 (58.3)	453 (60.3)	225 (54.5)	51 (58.6)	
Alcohol in terms of binge drinking (n (%))					
Less than once a month	217 (17.3)	128 (17.0)	75 (18.3)	14 (15.7)	
At least once a month	239 (19.1)	138 (18.4)	86 (21.0)	15 (16.9)	0.011
At least once a week	185 (14.8)	94 (12.5)	79 (19.3) †	12 (13.5)	
Never	610 (48.8)	392 (52.1)	170 (41.5) †	48 (53.9)	
Recreational cannabis (n (%))					
Less than once a month	86 (6.9)	50 (6.6)	26 (6.3)	10 (11.2)	
At least once a month	26 (2.1)	15 (2.0)	11 (2.7)	0 (0.0)	0.389
At least once a week	65 (5.2)	36 (4.8)	23 (5.6)	6 (6.7)	
Never	1077 (85.9)	653 (86.6)	351 (85.4)	73 (82.0)	
Long-term health problems (n (%))					
Cancers ^c^	31 (2.4)	22 (2.9)	7 (1.7)	2 (2.2)	0.456
Pulmonary diseases ^d^	177 (13.9)	94 (12.2)	70 (16.9)	13 (14.6)	0.082
Cardiovascular diseases ^e^	261 (20.6)	150 (19.7)	85 (20.5)	26 (29.2)	0.108
Digestive, endocrine, and urinary diseases ^f^	159 (12.6)	97 (12.8)	52 (12.7)	10 (11.2)	0.919
Musculoskeletal conditions ^g^	264 (22.4)	157 (22.2)	82 (21.4)	25 (29.1)	0.293
Mental health conditions ^h^	276 (21.9)	165 (21.7)	89 (21.5)	22 (24.7)	0.795

Abbreviations used: N/n = number; SD = standard deviation; SLSJ = Saguenay-Lac-Saint-Jean; BMI = body mass index; CHSLD = Centre d’hébergement et de soins longue durée. ^a^ Proportion was calculated on available data for each variable. ^b^ Post-hoc comparison: symbols (‡ and †) indicate a subset of categories whose column proportions differ significantly from each other at the 0.5 level. ^c^ Includes all cancers and melanoma, excludes other types of skin cancer in the last 5 years. ^d^ Includes asthma, chronic bronchitis, emphysema, chronic obstructive pulmonary disease (COPD). ^e^ Includes stroke, angina, heart attack, auricular fibrillation, heart failure. ^f^ Includes bowel disease, liver disease, diabetes, renal failure. ^g^ Includes musculoskeletal disorders, arthritis or rheumatoid arthritis. ^h^ Includes mood and anxiety disorders.

**Table 2:** COVID-19 episode.

At Onset	Total N = 1349
Severity (n (%))	
Mild	1259 (94.8)
Moderate	53 (4.0)
Severe	16 (1.2)
Symptoms (n (%))	
Asymptomatic	159 (12.0)
Respiratory ^a^	718 (55.4)
Gastrointestinal ^b^	592 (45.8)
Musculoskeletal ^c^	568 (44.0)
Neurologic ^d^	890 (69.0)
Constitutional ^e^	946 (73.6)
ORL ^f^	837 (64.9)
Other assessed ^g^	285 (22.2)
Vaccination at episode (number of doses) (n (%))	
None	1131 (86.9)
One	143 (11.0)
Two	27 (2.1)
**At time of survey**	
Post-COVID-19 conditions	622 (48.0)
Fatigue	
FSS (mean ± SD)	3.3 ± 1.6
FSS (n (%) of ≥5)	234 (19.6)
SOFA (mean ± SD)	1.6 ± 0.6
SOFA (n (%) of ≥2)	266 (22.6)
FSS ≥ 5 and SOFA ≥ 2 (n (%))	158 (13.0)
Psychological distress (mean ± SD)	4.0 ± 4.0
Psychological distress (n (%) of ≥14)	44 (3.5)
Daily function (n (%) of no)	
DASI1	134 (10.7)
DASI2	108 (8.6)
DASI3	97 (7.9)
DASI4	354 (33.3)
Function (EQ-5D) (n (%) of no or slight problems)	
Ability to walk	1228 (95.0)
Ability to bathe and dress	1281 (99.0)
Ability to complete activities of daily life	1227 (94.9)
Having pain or discomfort	1151 (89.6)
Being anxious or depressive	1093 (85.0)
Being short of breath (n (%))	
Out of breath with intense exercise	889 (71.2)
Out of breath when rushing or climbing a slight incline	265 (21.2)
Slower than most people of the same age on flat ground	55 (4.4)
Stop to breathe when walking 100 m on flat ground	34 (2.7)
Too out of breath to leave the house	5 (0.4)
Ceased occupation (n (%))	
Two weeks	766 (62.0)
Three to four weeks	321 (26.0)
One to three months	89 (7.2)
More than three months	36 (2.9)
Ceased occupation	24 (1.9)
Changes to occupation (n (%))	
Same occupation, more demanding	118 (10.7)
Same occupation, same conditions	863 (77.8)
Same occupation, less demanding	43 (3.9)
Different occupation, more demanding	24 (2.2)
Different occupation, same conditions	30 (2.7)
Different occupation, less demanding	31 (2.8)
Practitioner or nurse practitioner (n (%))	
Practitioner	1047 (78.5)
Nurse practitioner	34 (2.6)
Both	24 (1.8)
None	228 (17.1)
Access to health professionals (n (%))	
Difficulties with appointment system	122 (17.4)
Professionals not available	118 (17.6)
Transportation problems	20 (3.0)

Abbreviations used: N/n = number, SD = standard deviation, FSS = Fatigue Severity Scale, SOFA = Schedule of Fatigue and Anergia/General Practice, DASI = Duke Activity Status Index, EQ-5D = EuroQol-5D. ^a^ Includes cough, sibilance, hissing, stridor. ^b^ Includes nausea/vomiting, dysphagia, diarrhea, abdominal pain. ^c^ Includes joint pain and lower limb edema. ^d^ Includes aphasia, dysarthria, confusion, convulsions, ageusia, dysgeusia, anosmia, paresthesia of the lower or upper limbs. ^e^ Includes fever, dizziness, inappetence. ^f^ Includes earache, sore throat, hemoptysis, nasal discharge or congestion. ^g^ Includes eye infection, chest pain, rash.

**Table 3:** Post-COVID-19 conditions.

At the Time of Survey	
	PCC N = 622 (48.0%)
Symptoms at onset (n (%))	
Respiratory ^a^	389 (62.9)
Gastrointestinal ^b^	353 (57.5)
Musculoskeletal ^c^	319 (52.0)
Neurologic ^d^	512 (83.5)
Constitutional ^e^	494 (80.9)
ORL ^f^	451 (73.7)
Other assessed ^g^	189 (31.2)
Symptoms at time of survey (n (%))	
Respiratory ^a^	176 (28.6)
Gastrointestinal ^b^	161 (26.2)
Musculoskeletal ^c^	270 (43.9)
Neurologic ^d^	366 (59.3)
Constitutional ^e^	176 (28.6)
ORL ^f^	195 (31.9)
Other assessed ^g^	128 (21.1)
Fatigue at the time of survey	
FSS (n (%) of ≥5)	181 (31.9)
SOFA (n (%) of ≥2)	205 (37.8)
FSS ≥ 5 and SOFA ≥ 2 (n (%))	131 (23.0)
Psychological distress at the time of survey (n (%) of ≥14)	36 (6.1)

Abbreviations used: N/n = number, FSS = Fatigue Severity Scale, SOFA = Schedule of Fatigue and Anergia/General Practice. ^a^ Includes cough, sibilance, hissing, stridor. ^b^ Includes nausea/vomiting, dysphagia, diarrhea, abdominal pain. ^c^ Includes joint pain and lower limb edema. ^d^ Includes aphasia, dysarthria, confusion, convulsions, ageusia, dysgeusia, anosmia, paresthesia of the lower or upper limbs. ^e^ Includes fever, dizziness, inappetence. ^f^ Includes earache, sore throat, hemoptysis, nasal discharge or congestion. ^g^ Includes eye infection, chest pain, rash.

**Table 4:** Post-COVID-19 condition characteristics.

At Time of Onset					
	No PCC and FSS < 5 N = 571 (47.9)	PCC and FSS < 5 N = 387 (32.5)	No PCC and FSS ≥ 5 N = 53 (4.4)	PCC and FSS ≥ 5 N = 181 (15.2)	*p*-Value
Age	45.8 ± 15.1	48.2 ± 16.9	43.0 ± 16.3	48.7 ± 15.0	0.010
Sex (Female)	288 (51.8)	226 (59.8)	34 (66.7)	120 (68.2)	<0.001
BMI	27.1 ± 5.5	28.1 ± 5.6	27.4 ± 5.4	29.0 ± 6.3	0.004
Pulmonary diseases	48 (8.5)	59 (15.4)	6 (11.8)	48 (26.8)	<0.001
Cardiovascular diseases	92 (16.3)	79 (20.7)	13 (24.5)	57 (32.0)	<0.001
Musculoskeletal conditions	74 (14.3)	94 (25.9)	12 (26.7)	69 (40.4)	<0.001
Mental health conditions	77 (13.7)	82 (21.4)	21 (40.4)	80 (44.9)	<0.001
Severity of episode					<0.001
Mild	551 (96.7)	362 (94.3)	51 (98.1)	159 (88.3)	
Moderate or severe	19 (3.3)	22 (5.7)	1 (1.9)	21 (11.7)	
Number of symptoms at onset	4.9 ± 3.9	7.1 ± 4.3	6.5 ± 4.9	9.6 ± 4.2	<0.001

**Table 5:** Post-COVID-19 condition risk factors.

At Time of Onset			
PCC and FSS < 5 N = 387 (32.5)			
	OR	95% CI	*p*-Value
Age	1.010	1.002–1.018	0.020
Sex (Female)	1.383	1.062–1.802	0.016
BMI	1.029	1.003–1.058	0.028
Pulmonary diseases	1.965	1.308–2.941	0.001
Cardiovascular diseases	1.340	0.961–1.873	0.085
Musculoskeletal conditions	2.100	1.495–2.950	<0.001
Mental health conditions	1.715	1.218–2.415	0.002
Severity of episode	1.764	0.941–3.300	0.077
Number of symptoms	1.136	1.098–1.176	<0.001
Being a health professional	1.038	0.752–1.434	0.819
PCC and FSS ≥ 5 N = 181 (15.2)			
	OR	95% CI	*p*-Value
Age	1.012	1.001–1.023	0.029
Sex (Female)	1.996	1.395–2.849	<0.001
BMI	1.056	1.024–1.090	<0.001
Pulmonary diseases	3.968	2.545–6.173	<0.001
Cardiovascular diseases	2.421	1.647–3.559	<0.001
Musculoskeletal conditions	4.065	2.747–6.024	<0.001
Mental health conditions	5.155	3.509–7.519	<0.001
Severity of episode	3.831	2.008–7.299	<0.001
Number of symptoms	1.292	1.234–1.352	<0.001
Being a health professional	1.525	1.024–2.270	0.038

## Data Availability

The data presented in this study are available on request from the corresponding author. The data are not publicly available due to ethical and privacy issues.

## Supplementary Materials

The following supplementary information can be downloaded at: https://www.mdpi.com/article/10.3390/jcm12185939/s1. Supplementary S1 details sample size calculation and randomization. Supplementary S2 presents the complete questionnaires in English and French.
