# Supplementary material for: Long-Term Consequences of COVID-19 in Predominantly Immunonaive Patients: A Canadian Prospective Population-Based Study
Source: J Clin Med. 2023 Sep 13;12(18):5939. doi: 10.3390/jcm12185939 (PMC10532279; doi:10.3390/jcm12185939)
Supplement: Supplementary file 1 [file jcm-12-05939-s001.zip › jcm-2459369-supplementary.pdf]

supplementary S1 – Sample size calculation and randomization

A total of 900 to 1,200 participants (i.e., 300 to 400 per study region) having completed the questionnaire were required considering 10 events per variable, between 6 and 8 variables, a binary outcome, and an intraclass coefficient of 20%.

A stratified block randomization was used to select a sample of individuals with SARS-CoV-2 proven infection from each of the three study regions. We completed the random selection in multiple steps, as outlined below.

Firstly, all subjects with a positive PCR test between November 1, 2020, and May 31, 2021, were divided by their region: Estrie, Saguenay-Lac-Saint-Jean, or New Brunswick. We created eight subsequent blocks for each region by age range (18-29, 30-39, 40-49, 50-59, 60-69, 70-79, 80-89, and 90 or older).

The second step was determining the number of participants per region and age range needed. To do this, we considered the proportion of participants in each age range between the three regions. The number of participants required was calculated based on this proportion to have an even representation of each age range and region.

A number was assigned to every participant and randomly reorganized in Microsoft Excel. The first participants corresponding to the required number per region and age range were selected.

supplementary S2 - Questionnaires

Questionnaire – English version

02. QUESTIONNAIRE

The following questions touch upon several themes. Topics will therefore change several times throughout the survey.

SET 1 - DEMOGRAPHIC

HERE IS THE FIRST SET OF QUESTIONS

TC1A **Which language do you speak most often at home?**

1 French → *FILTER A: GO TO TC1B*

2 English

3 Other → *FILTER A*

8 Doesn't know

9 Doesn't answer

FILTER A: IF ANSWER TO TC1A IS FRENCH (01) OR OTHER (03), GO TO TC1B. OTHERWISE, GO TO TC2.

TC1B **Would you prefer to answer the French version of the questionnaire?**

1 Yes

2 No

END OF FILTER A.

TC2 **In what year were you born?**

Year in AAAA format : ____________.

TC2A **How old are you?**

TC3 **What are the first three characters of your postal code?**

TC4 **What type of environment do you live in?**

1 Private household

2 Apartment

3 Private residence for seniors

4 CHSLD

5 Intermediate and family-type resources

6 Rooming house
7 Other
98 Doesn't know
99 Doesn't answer

TC6A **How many people aged 17 and younger live in your household at least half of the time?**

0 None → *FILTER B: GO TO TC6C*
1 One → *FILTER C: GO TO TC6B*
2 Two → *FILTER C*
3 Three → *FILTER C*
4 Four → *FILTER C*
5 Five → *FILTER C*
6 Six → *FILTER C*
7 Seven → *FILTER C*
8 Eight → *FILTER C*
9 Nine → *FILTER C*
10 Ten and more → *FILTER C*
98 Doesn't know..98
99 Doesn't answer...99

FILTER B IF THE ANSWER TO TC6A IS NONE (0), GO TO TC6C.
FILTER C IF THE ANSWER TO TC6A IS ONE (01) TO TEN,(10) GO TO TC6B.

TC6B **How many people aged 5 and under live in your household at least half of the time?**

0 None
1 One
2 Two
3 Three
4 Four
5 Five and more
98 Doesn't know
99 Doesn't answer

END FILTER C.

TC6C **How many people aged 18 and over, including you, live in your household at least half the time?**

1 One → *FILTER D: GO TO TC6D*
2 Two→ *FILTER D*
3 Three → *FILTER D*
4 Four → *FILTER D*
5 Five → *FILTER D*
6 Six → *FILTER D*
7 Seven → *FILTER D*
8 Eight → *FILTER D*
9 Nine → *FILTER D*
10 Ten and more → *FILTER D*
98 Doesn't know
99 Doesn't answer

END FILTER B.

FILTER D: IF THERE ARE AT LEAST TWO PEOPLE AGED 18 AND MORE (02) TO (10) IN TC6C OR 1 ADULT (01) IN TC6C AND 1 TO 10 PEOPLE AGED 17 AND UNDER AT LEAST HALF THE TIME (TC6A: 01 – 10), GO TO TC6D OTHERWISE CONTINUE WITH TC8A.

TC6D **Who lives with you in your home?**

INTERVIEWER'S GUIDELINE: Read the choices or help the participant by telling him/her to consider all members of the household.

1 Couple (with or without children at home)
2 Single-parent family (with children of any age)
3 Other (unrelated people, co-tenants, subletter, etc)
98 Doesn't know
99 Doesn't answer

END FILTER D.

TC8A **During the past 12 months, what was your main occupation?**

INTERVIEWER'S GUIDELINE: Read the choices, if needed.

0 Self-employed → *FILTER E: GO TO TC8B*

1 Full-time worker → *FILTER E*

2 Part-time worker → *FILTER E*

3 Student

4 Retired

5 Semi-retired → *FILTER E*

6 Homemaker

7 On maternity/paternity leave

8 Employment insurance recipient

9 Social aid (income security) recipient

10 Sick leave/CSST

11 Disability/SAAQ

12 Seasonal worker

97 Other

98 Doesn't know

99 Doesn't answer

TC8AA **If other (97) to TC8A, please specify: ___________________________.**

FILTER E: IF PARTICIPANT IS SELF-EMPLOYED (00), FULL-TIME WORKER (01), PART-TIME WORKER (02), OR SEMI-RETIRED (13) TO TC8A, GO TO TC8B, OTHERWISE CONTINUE TO NEXT SET.

TC8AAA **Have you had a paid job in the past two years?**

1 Yes → GO TO TC8B

2 No → GO TO next set

9 Doesn't answer

TC8B **Do you work in the health and social services area?**

1 Yes → GO TO TC8B1

2 No

9 Doesn't answer

END FILTER E.

TC8B1 **Do you work, or have you worked in direct contact with patients ?**

1 Yes

2 No

9 Doesn't answer

TC8B4 **How long have you been off your job because of the lockdown and other restrictions imposed by the government?**

1 I didn't stop working because of the lockdown
2 Less than a week
3 One to two weeks
4 Three to four weeks
5 One to three months
6 Three to six months
7 More than six months
8 I did not return to my job
98 Doesn't know
99 Doesn't answer

TC8B5 **How long have you been off your job because of your COVID-19 episode?**

1 I didn't stop working because of my COVID-19 episode, except for the two weeks of confinement
2 Three to four weeks
3 One to three months
4 Three to six months
5 More than six months
6 I did not return to my job
98 Doesn't know
99 Doesn't answer

TC8B6 **Have you had to change jobs or change your job following your COVID-19 episode?**

1 I have the same job on a more demanding workload
2 I have the same job with a similar workload
3 I have the same job on a less demanding workload
4 I changed my job for one with a more demanding workload
5 I changed my job for a job with the same workload
6 I changed my job for a job with a less demanding workload
98 Doesn't know
99 Doesn't answer

SET 2 – COVID-19 EPISODE

THE NEXT SECTION IS ABOUT YOUR COVID-19 EPISODE

COVID1 **What was the degree of severity of your COVID-19 episode?**

**Mild**	1 Asymptomatic
	2 Symptomatic
**Moderate**	3 Hospitalized without oxygen
	4 Hospitalized with oxygen
**Severe**	5 Hospitalized in intensive care
	6 Hospitalized with a ventilator (intubated))
	98 Doesn't know
	99 Doesn't answer

TC11A_Acces **Do you have access to a family doctor or a nurse practitioner?**

0 Yes, I have a nurse practitioner
1 Yes, I have a family doctor
2 Yes, I have a family doctor and a nurse practitioner
3 No
98 Doesn't know
99 Doesn't answer

COVID2 **Since your episode of COVID-19, how many outpatient appointments have you had in connection with symptoms potentially related to COVID-19 with each of these professionals?**

A Doctor
B Nurse
C Physical therapist
D Occupational therapist
E Psychologist or another mental health professional
(Choice, 0, 1, 2, 3, 4, 5+) → GO TO COVID2A if COVID2≥1 for at least one professional.

COVID2A **How long have you waited before seeing this professional between your episode of COVID-19 and the first appointment?**

A Doctor
B Nurse
C Physical therapist
D Occupational therapist
E Psychologist or another mental health professional
Choices :

0) No appointment with this professional
1) Less than a week
2) One to four weeks
3) One to three months
4) Three to six months
5) Over six months

COVID3 **What types of professionals do you think you need follow-up with for symptoms related to your COVID-19 episode that you haven't been able to get yet?**

A Doctor
B Nurse
C Physical therapist
D Occupational therapist
E Psychologist or another mental health professional
(multiple choices available)

COVID4 **Which of the following are some of the difficulties you have experienced in obtaining care?**

Answer yes or no for the following difficulties.

	Yes (01)	No (02)	Doesn't apply (07)	Doesn't answer (09)
**Difficulties with the appointment system?**				
**Professionals not available (no appointment)?**				
**Transportation problems?**				

COVID5 **Has having had an episode of COVID-19 changed your compliance with health measures?**

1 More respectful of sanitary measures
2 Unchanged
3 Less respectful of sanitary measures
8 Doesn't know
9 Doesn't answer

COVID6 **Has having COVID-19 changed your opinion on vaccination against COVID-19?**

1 More inclined to receive the vaccine

2 Unchanged

3 Less inclined to receive the vaccine

8 Doesn’t know

9 Doesn’t answer

COVID7 **In your opinion, are COVID-19 vaccines effective?**

1 Not at all

2 A little

3 Enough

4 Very

8 Doesn’t know

9 Doesn’t answer

COVID8 **In your opinion, are COVID-19 vaccines safe?**

1 Not at all

2 A little

3 Enough

4 Very

8 Doesn’t know

9 Doesn’t answer

COVID9 **In your opinion, did you receive enough information about these vaccines to decide whether or not to get vaccinated (informed consent)?**

1 Not at all

2 A little

3 Enough

4 Very

8 Doesn’t know

9 Doesn’t answer

COVID10 **Have you received a COVID-19 vaccine?**

0 No

1 1 shot before your COVID-19 episode

2 2 shots before your COVID-19 episode

3 1 shot after your COVID-19 episode

4 2 shots after your COVID-19 episode

8 Doesn’t know

9 Doesn’t answer

**ACC _COVID1**	**Excluding the first month after your COVID-19 diagnosis, did you need to go to the hospital urgently (unexpectedly)?**<br>1 Yes → GO TO ACC_COVID2<br>2 No → GO TO SY_COVID1<br>98 Doesn’t know<br>99 Doesn’t answer
**ACC _COVID2**	**Where in the hospital did you receive care?**<br>1 Admitted to hospital ward<br>2 Emergency room/Outpatient clinic<br>98 Doesn’t know<br>99 Doesn’t answer

Which of the following symptoms did you experience when you were first diagnosed with COVID-19?

**SY_COVID1**	**Confusion ?**	1 Yes<br>2 No<br>98 Doesn’t know<br>99 Doesn’t answer
**SY_COVID2**	**Convulsions?**	
**SY_COVID3**	**Fever?**	
**SY_COVID4**	**Speech disturbance/Difficulty speaking?**	

Which of the following symptoms did you experience during your initial episode of COVID-19, and which are you still experiencing to this day?

**SY_COVID5**	**Joint pain**	0 No<br>1 Yes, gone<br>2 Yes, always the same<br>3 Yes, increased<br>4 Yes, decreased<br>5 Appeared more than a month after my COVID-19 episode<br>98 Doesn’t know<br>99 Doesn’t answer
**SY_COVID6**	**Stomach aches?**	
**SY_COVID7**	**Chest pain?**	
**SY_COVID8**	**Earache?**	
**SY_COVID9**	**Sore throat?**	
**SY_COVID10**	**Cough?**	
**SY_COVID11**	**Coughing up blood?**	
**SY_COVID12**	**Runny or stuffy nose?**	

**SY_COVID13**	**Loud breathing?**	
**SY_COVID14**	**Dizziness?**	
**SY_COVID15**	**Weakness or numbness in the legs or arms?**	
**SY_COVID16**	**Swelling of the legs?**	
**SY_COVID17**	**Loss of smell?**	0 No<br>1 Yes, gone
**SY_COVID18**	**Loss of taste?**	2 Yes, always the same<br>3 Yes, increased
**SY_COVID19**	**Alteration of taste?**	4 Yes, decreased<br>5 Appeared more than a month
**SY_COVID20**	**Loss of appetite?**	after my COVID-19 episode<br>98 Doesn't know
**SY_COVID21**	**Nausea / vomiting?**	99 Doesn't answer
**SY_COVID22**	**Difficulty swallowing liquids or foods?**	
**SY_COVID23**	**Diarrhea?**	
**SY_COVID24**	**Eye infection?**	
**SY_COVID25**	**Rash**	

We are now going to ask you some questions about how you function in your activities of daily living.

**FCT _COVID1**	**Which statement best describes your mobility?**<br>1 I have no problem walking<br>2 I have mild problems walking<br>3 I have moderate problems walking<br>4 I have severe problems walking<br>5 I cannot walk<br>98 Doesn't know<br>99 Doesn't answer
**FCT _COVID2**	**Which statement best describes your ability to complete your personal care?**

1 I have no problem to wash and dress
2 I have mild problems to wash and dress
3 I have moderate problems to wash and dress
4 I have severe problems to wash and dress
5 I cannot wash and dress
98 Doesn’t know
99 Doesn’t answer

FCT _COVID3 **Which statement best describes your ability to complete your usual activities**
1 I have no problem to complete my usual activities
2 I have mild problems to complete my usual activities
3 I have moderate problems to complete my usual activities
4 I have severe problems to complete my usual activities
5 I cannot complete my usual activities
98 Doesn’t know
99 Doesn’t answer

FCT _COVID4 **Which statement best describes your pain and discomfort?**
1 I have no pain or discomfort
2 I have mild pain or discomfort
3 I have moderate pain or discomfort
4 I have severe pain or discomfort
5 I have extreme pain or discomfort
98 Doesn’t know
99 Doesn’t answer

FCT _COVID5 **Which statement best describes your anxiety and depression?**
1 I am not anxious or depressed
2 I am mildly anxious or depressed
3 I am moderately anxious or depressed
4 I am severely anxious or depressed
5 I am extremely anxious or depressed
98 Doesn’t know
99 Doesn’t answer

FCT _COVID6 **Which statement best describes your shortness of breath?**
1 I have shortness of breath only with intense exercise
2 I am out of breath rushing or going up a slight incline
3 I am slower than most people of the same age on flat ground
4 I stop to breathe while walking 100 meters or a few minutes on flat ground
5 I am too short of breath to leave the house

98 Doesn’t know
99 Doesn’t answer

The next few questions relate to your fatigue. Subsequent statements will be evaluated on a scale varying from strongly disagree to strongly agree.

In the past week, I found that…

FSS1 **My motivation is lower when I am fatigued.**
1 Strongly disagree
2 Disagree
3 Somewhat disagree
4 Neutral
5 Somewhat agree
6 Agree
7 Strongly agree
8 Doesn’t know
9 Doesn’t answer

FSS2 **Exercise brings on my fatigue.**
1 Strongly disagree
2 Disagree
3 Somewhat disagree
4 Neutral
5 Somewhat agree
6 Agree
7 Strongly agree
8 Doesn’t know
9 Doesn’t answer

FSS3 **I am easily fatigued**
1 Strongly disagree
2 Disagree
3 Somewhat disagree
4 Neutral
5 Somewhat agree
6 Agree
7 Strongly agree

8 Doesn’t know
9 Doesn’t answer

FSS4 **Fatigue interferes with my physical functioning.**
1 Strongly disagree
2 Disagree
3 Somewhat disagree
4 Neutral
5 Somewhat agree
6 Agree
7 Strongly agree
8 Doesn’t know
9 Doesn’t answer

FSS5 **Fatigue causes frequent problems for me.**
1 Strongly disagree
2 Disagree
3 Somewhat disagree
4 Neutral
5 Somewhat agree
6 Agree
7 Strongly agree
8 Doesn’t know
9 Doesn’t answer

FSS6 **My fatigue prevents sustained physical functioning.**
1 Strongly disagree
2 Disagree
3 Somewhat disagree
4 Neutral
5 Somewhat agree
6 Agree
7 Strongly agree
8 Doesn’t know
9 Doesn’t answer

FSS7 **Fatigue interferes with carrying out certain duties and responsibilities.**
1 Strongly disagree
2 Disagree
3 Somewhat disagree

4 Neutral
5 Somewhat agree
6 Agree
7 Strongly agree
8 Doesn’t know
9 Doesn’t answer

FSS8 **Fatigue is among my most disabling symptoms.**
1 Strongly disagree
2 Disagree
3 Somewhat disagree
4 Neutral
5 Somewhat agree
6 Agree
7 Strongly agree
8 Doesn’t know
9 Doesn’t answer

FSS9 **Fatigue interferes with my work, family, or social life.**
1 Strongly disagree
2 Disagree
3 Somewhat disagree
4 Neutral
5 Somewhat agree
6 Agree
7 Strongly agree
8 Doesn’t know
9 Doesn’t answer

Please answer the next questions considering the past few weeks. The next statements will be evaluated on a scale varying from none or a little, to most of the time

SOFA1 **I feel tired for a long time after physical activity.**
1 None or little
2 Some of the time
3 Good part of the time
4 Most of the time
8 Doesn’t know
9 Doesn’t answer

SOFA2 **My concentration is poor.**
1 None or little
2 Some of the time

3 Good part of the time
4 Most of the time
8 Doesn't know
9 Doesn't answer

SOFA3 **My muscles feel very tired after physical activity.**
1 None or little
2 Some of the time
3 Good part of the time
4 Most of the time
8 Doesn't know
9 Doesn't answer

SOFA4 **I get headaches.**
1 None or little
2 Some of the time
3 Good part of the time
4 Most of the time
8 Doesn't know
9 Doesn't answer

SOFA5 **I need to sleep for long periods.**
1 None or little
2 Some of the time
3 Good part of the time
4 Most of the time
8 Doesn't know
9 Doesn't answer

SOFA6 **I get muscle pain after physical activity.**
1 None or little
2 Some of the time
3 Good part of the time
4 Most of the time
8 Doesn't know
9 Doesn't answer

SOFA7 **I sleep poorly.**

1 None or little
2 Some of the time
3 Good part of the time
4 Most of the time
8 Doesn't know
9 Doesn't answer

SOFA8 **I have problems with my speech (e.g. feeling "lost for word").**
1 None or little
2 Some of the time
3 Good part of the time
4 Most of the time
8 Doesn't know
9 Doesn't answer

SOFA9 **My memory is poor.**
1 None or little
2 Some of the time
3 Good part of the time
4 Most of the time
8 Doesn't know
9 Doesn't answer

SOFA10 **I get muscle pain even at rest.**
1 None or little
2 Some of the time
3 Good part of the time
4 Most of the time
8 Doesn't know
9 Doesn't answer

SET 3 – LONG TERM HEALTH PROBLEMS

THE NEXT QUESTIONS ARE RELATED TO LONG TERM HEALTH PROBLEMS YOU MAY HAVE.

By « long term health problems » we mean a problem that lasts or should last 6 months or more which was diagnosed by a health professional.

Interviewer guideline : if the answer is yes, ask if the condition is worst, better or identical since the episode of COVID-19 or if it is a new diagnosis.

Do you suffer from […]

		No (01)	Yes (02)	Worst since COVID (03)	Better since COVID (04)	Same since COVID (05)	New DX (06)
TC18A _SantéLD	Cancer (including melanoma and excluding other types of skin cancer) in the last 5 years ?						
TC18B _SantéLD	Pulmonary problems (asthma, chronic bronchitis, emphysema, chronic obstructive pulmonary disease (COPD)) ?						
TC18C _SantéLD	Stroke, even minor ?						
TC18D _SantéLD	Heart disease (angina, heart attack, atrial fibrillation) ?						
TC18E _SantéLD	Heart failure ?						
TC18F _SantéLD	Bowel disease (Crohn's disease, ulcerative colitis, diverticulosis) ?						
TC18G	Liver problems ?						

_SantéLD	
TC18H _SantéLD	Renal failure ?

Remember: we are interested in health problems diagnosed by a health professional, especially if you are taking medications for these conditions.

Do you suffer from […]

		No (01)	Yes (02)	Worst since COVID (03)	Better since COVID (04)	Same since COVID (05)	New DX (06)
TC18I _SantéLD	Musculoskeletal problems causing pain or limitations ?						
TC18J _SantéLD	Arthritis or rhumatoid arthritis ?						
TC18K _SantéLD	Diabetes ?						
TC18L _SantéLD	Hypertension ?						
TC18N _SantéLD	Mood disorder such as depression, bipolar disorder or mania ?						
TC18O _SantéLD	Anxiety disorder ?						

TC18P _SantéLD **Automatic calculation of the long-term illness score.**

EXPLANATIONS TO THE INTERVIEWER : A long-term illness score of 0 is indicative of better health than a score of 15, which corresponds to a positive response to all of the medical conditions listed.

SET 4 – LIFESTYLE

LET'S CONTINUE WITH QUESTIONS ABOUT LIFESTYLE.

TC21 _FetL **How many fruits, vegetables or portions of these do you eat each day?**

1 Five or more

2 Four

3 Three

4 Two

5 Less than two

8 Doesn't know

9 Doesn't answer

TC22 _Habitudes **In your lifetime, have you smoked at least one hundred cigarettes or more(approximately 4 packs) ?**

1 Yes

2 No

98 Doesn't know

99 Doesn't answer

TC23 _Habitudes **At the present time, how often do you smoke tobacco (including cigarettes, cigars, cigarillos and pipes)?**

1 Everyday

2 Occasionally

3 Never

98 Doesn't know

99 Doesn't answer

TC16A _FetL **How tall are you without shoes on?**

Height, value in inches : ________________.

If doesn't know or doesn't answer, indicate « NA ». Value must be comprised between 41 and 89 inches.

TC16B _FetL **How much do you weigh ?**

Weight, value in pounds: ________________.

If doesn't know or doesn't answer, indicate « NA ». Value must be comprised between 75 and 550 pounds.

TC16C _FetL ***Automatic calculation of BMI.***

SET 5 – ALCOOL AND DRUG CONSUMPTION

THE NEXT QUESTIONS CONCERN THE CONSUMPTION OF ALCOHOL AND DRUGS

TC22_Consom **Since your COVID-19 episode, how often did you drink 5 or more glasses of alcohol on the same occasion?**

1 Never
2 Less than once a month
3 Once a month
4 2 to 3 times a month
5 Once a week
6 More than once a week
8 Doesn’t know
9 Doesn’t answer

TC22A_Consom **Since your COVID-19 episode, how has your alcohol consumption evolved?**

1 I stopped consuming it
2 I consume much less than before
3 I consume less than before
4 I consume the same amount as before
5 I consume more than before
6 I consume much more than before
8 Doesn’t know
9 Doesn’t answer

TC23_Consom **Since your COVID-19 episode, how frequently have you used cannabis (marijuana, hashish, hashish oil or other by-products) for recreational purposes?**

1 Never
2 Once or twice
3 3 to 11 times a year
4 1 to 3 times a month
5 Once a week
6 More than once a week
8 Doesn’t know
9 Doesn’t answer

TC23A_Consom **Since your COVID-19 episode, how has your cannabis use evolved?**

1 I stopped consuming it
2 I consume much less than before
3 I consume less than before
4 I consume the same amount as before

5 I consume more than before

6 I consume much more than before

8 Doesn’t know

9 Doesn’t answer

SET 6 – CARDIOVASCULAR CAPACITIES

THE NEXT QUESTIONS CONCERN PHYSICAL ACTIVITY

DASI1 **Are you able to climb a flight of stairs or walk up a hill ?**

1 Yes
2 No
8 Doesn’t know
9 Doesn’t answer

DASI2 **Are you able to do heavy house work around the house like scrubbing floors or lifting or moving heavy furniture ?**

1 Yes
2 No
8 Doesn’t know
9 Doesn’t answer

DASI3 **Are you able to do yard work like raking leaves, weeding or pushing a power mower ?**

1 Yes
2 No
8 Doesn’t know
9 Doesn’t answer

DASI4 **Are you able to participate in strenuous sports like swimming, singles tennis, football, basketball or skiing ?**

1 Yes
2 No
8 Doesn’t know
9 Doesn’t answer

SET 7 – PSYCHOLOGICAL IMPACTS OF COVID-19

THE NEXT QUESTIONS ARE ABOUT THE PSYCHOLOGICAL IMPACT OF CORONAVIRUS.

CONF1 **In general, how do you currently find your social life, i.e. your relationships with the people around you (parents, friends, acquaintances)?**

1 Very satisfactory
2 Rather satisfactory
3 Rather unsatisfactory
4 Very unsatisfactory
8 Doesn't know
9 Doesn't't' answer

In the last month, how often did you felt […]

INTERVIEWER'S GUIDELINE : Read the choices.

		Never (00)	Rarely (01)	Sometimes (02)	Most of the time (03)	Always (04)
TC19A _VieQuo	Nervous					
TC19B _VieQuo	Desperate					
TC19C _VieQuo	Restless or not able to stay still					
TC19D _VieQuo	So depressed that nothing could make you smile					
TC19E _VieQuo	Good for nothing					
TC19F _VieQuo	That everything was an effort					

Doesn't know..98
Doesn't answer..99

TC19G _VieQuo ***Automatic calculation of the psychological distress index.***

EXPLANATIONS TO THE INTERVIEWER :
The psychological distress index ranges from 0 to 24. The higher the index, the higher is the level of psychological distress.

Remember that all the information you provide will be kept strictly confidential.

VC1 **In connection with your marital status, since your episode of COVID-19, is the situation in the marital relationship…**

1 Much better
2 A little better
3 Stayed the same
4 A little worse
5 Much worse → GO TO VC2
08 Doesn't know
09 Doesn't answer

VC2 **Do you feel the need for me to give you resource contacts to talk to someone about this situation ?**

Resources :

L'Escale de l'Estrie
www.escaleestrie.com
819-569-3611 (Service 24/7)

La Méridienne (Estrie)
www.lameridienne.ca
1-888-699-3050

Centre féminin du Saguenay
418-549-4343

Carrefour pour femmes (Moncton)
506-875-5315

Centre de ressources et de crises familiales Beauséjour (Shediac)

506-533-9100

L’Accueil Sainte-Famille Inc. (Tracadie)
506-395-1500

SET 8 – ANXIETY AND MANAGEMENT STRATEGIES

THE NEXT SECTION IS ABOUT ANXIETY AND MANAGEMENT STRATEGIES.

STR1 **If you compare your anxiety level today with that before your COVID-19 episode, would it be…**

1 Higher
2 Lower
3 The same
8 Doesn't know
9 Doesn't answer

STR2 **What are you doing to decrease your anxiety and stress?**

1 Physical activity
2 Cultural activity
3 Alcohol consumption
4 Drugs
5 Social activity
6 Consultation of mental health professional
7 Spirituality
8 Relaxation
9 Other
10 None
98 Doesn't know
99 Doesn't answer

SET 9 – CAREGIVER

THIS SECTION IS ABOUT CAREGIVERS.

PRA1A **Are you a caregiver?**

1 Yes → GO TO PRA1B

2 No → GO TO PRA2A

8 Doesn't know

9 Doesn't answer

PRA1B **Did your COVID-19 episode prevent you from fulfilling your role as a caregiver?**

1 Yes, during lockdown

2 Yes, I was not able to resume my role at its usual level

3 No

8 Doesn't know

9 Doesn't answer

PRA2A **Do you receive help from a caregiver?**

1 Yes → GO TO PRA2B

2 No → GO TO NEXT SET

8 Doesn't know

9 Doesn't answer

PRA2B **Is the need for this help related to your COVID-19 episode?**

1 Yes

2 No

8 Doesn't know

9 Doesn't answer

SET 10 – NEWS

THE FOLLOWING QUESTIONS ARE BOUT THE NEWS AND THE CORONAVIRUS PANDEMIC

NOUV1A **On a scale from 1 to 10, do you find the media adequately reports what it is like to have COVID-19?**

1 Complete disagreement

…

10 Complete agreement

97 I don't listen to media

98 Doesn't know

99 Doesn't answer

To what extent do you agree with the following statements regarding the coronavirus pandemic?

NOUV2A **On a scale from 1 to 10, before my COVID-19 episode, I felt that I already had all the information I needed to fully understand the coronavirus.**

1 Complete disagreement

…

10 Complete agreement

97 I don't listen to the media

98 Doesn't know

99 Doesn't answer

NOUV2B **On a scale from 1 to 10, in my experience, once you have had the coronavirus, you can have lifelong damage.**

1 Complete disagreement

…

10 Complete agreement

97 I don't listen to the media

98 Doesn't know

99 Doesn't answer

SET 11 – DEMOGRAPHIC 2

THIS IS THE LAST SET OF QUESTIONS.

TC4A **Were you born in Canada?**

1 Yes

2 No → FILTER F : GO TO TC4B

8 Doesn't know

9 Doesn't answer

FILTER F : IF NO (02) TO TC4A, GO TO TC4B, OTHERWISE GO TO TC3B.

TC4B **How many years have you lived in Canada?**

1 4 years or less

2 5 years or more

8 Doesn't know

9 Doesn't answer

END FILTER F.

TC3B **What sex were you assigned at birth?**

1 Male

2 Female

9 Doesn't answer

TC3A_2 **Does this sex match your gender (by gender, we mean the current gender, which may differ from sex assigned at birth or from that recorded in legal documents)?**

1 Yes

2 No

8 Doesn't know

9 Doesn't answer

OS1 **What is your sexual orientation?**

1 Heterosexual

2 Homosexual

3 Bisexual

4 Other

8 Doesn't know

9 Doesn't answer

TC9A **What is the highest level of formal education you have completed?**

1 No certificate or diploma

2 High school diploma or equivalent

3 Vocational or trade school diploma or certificate

4 Cegep certificate or diploma or other non university diploma

5 University certificate or diploma

6 Other →GO TO TC9AA

8 Doesn't know

9 Doesn't answer

TC9AA **If other (97) to TC9A, please specify : ___________________.**

TC7 **What was the total approximate income before taxes for your household in 2020 (ex : work, social welfare, unemployment benefit, pension plan) ?**

1 Less than 19 999$
2 20 000$ to 29 999$
3 30 000$ to 49 999$
4 50 000$ to 69 999$
5 70 000$ to 89 999$
6 90 000$ to 149 999$
7 More than 150 000$
8 Doesn't know
9 Doesn't answer

02. QUESTIONNAIRE

Je vous informe que les questions portent sur plusieurs thèmes, les sujets changeront donc à quelques reprises dans le questionnaire.

BLOC 1 - GÉNÉRALITÉS

COMMENÇONS PAR LE PREMIER BLOC DE QUESTIONS

TC1A **Quelle langue parlez-vous le plus souvent à la maison ?**

1 Français
2 Anglais → *FILTRE A : ALLEZ À LA TC1B*
3 Autre → *FILTRE A*
8 Ne sait pas
9 Ne répond pas

FILTRE A : SI RÉPONSES ANGLAIS (02) OU AUTRE (03) À LA TC1A, ALLEZ À LA TC1B. SINON, POURSUIVEZ À LA TC2

TC1B **Préférez-vous répondre à la version anglophone du questionnaire ?**

1 Oui
2 Non

FIN FILTRE A.

TC2 **Quelle est votre année de naissance ?**

Année en format AAAA : ____________.

TC2A **Quel est votre âge ?**

TC3 **Quels sont les 3 premiers caractères de votre code postal ?**

TC4 **Dans quel type de milieu habitez-vous ?**

1 Résidence privée
2 Appartement
3 Résidence privée pour aînés
4 CHSLD
5 Ressources intermédiaires et de type familiale
6 En maison de chambres
7 Autre

98 Ne sait pas
99 Ne répond pas

TC6A **Combien de personnes de 17 ans et moins habitent dans votre foyer au moins la moitié du temps ?**

0 Aucune → *FILTRE B : ALLEZ À LA TC6C*
1 Une→ *FILTRE C : ALLEZ À LA TC6B*
2 Deux → *FILTRE C*
3 Trois → *FILTRE C*
4 Quatre → *FILTRE C*
5 Cinq → *FILTRE C*
6 Six → *FILTRE C*
7 Sept → *FILTRE C*
8 Huit → *FILTRE C*
9 Neuf → *FILTRE C*
10 Dix et plus → *FILTRE C*
98 Ne sait pas
99 Ne répond pas

FILTRE B : SI RÉPONSES AUCUNE (0), ALLEZ À LA T6C.
FILTRE C : SI RÉPONSES UNE (01) À DIX (10) À LA TC6A, ALLEZ À LA T6B, SINON POURSUIVEZ À LA TC6C.

TC6B **Combien de personnes de 5 ans et moins habitent dans votre foyer au moins la moitié du temps ?**

0 Aucune
1 Une
2 Deux
3 Trois
4 Quatre
5 Cinq et plus
98 Ne sait pas
99 Ne répond pas

FIN FILTRE C.

TC6C **Combien de personnes de 18 ans et plus, en vous incluant, habitent dans votre foyer ?**

1 Une → *FILTRE D : ALLEZ À LA TC6D*
2 Deux → *FILTRE D*
3 Trois → *FILTRE D*
4 Quatre → *FILTRE D*
5 Cinq → *FILTRE D*
6 Six → *FILTRE D*
7 Sept → *FILTRE D*
8 Huit → *FILTRE D*
9 Neuf → *FILTRE D*
10 Dix et plus → *FILTRE D*
98 Ne sait pas
99 Ne répond pas

FILTRE D : SI À LA TC6C, ON RETROUVE AU MOINS DEUX PERSONNES ÂGÉES DE 18 ANS ET PLUS DANS LE MÉNAGE (02) À (10) OU BIEN LA COMBINAISON SUIVANTE : 1 SEUL ADULTE (01) À LA TC6C ET 1 PERSONNE OU PLUS DE 17 ANS OU MOINS (TC6A : 01 – 10), ALLEZ À LA TC6D, SINON POURSUIVEZ À LA TC8A.

FIN FILTRE B.

TC6D **Quel type de situation correspond le mieux à la composition de votre foyer (ménage) ?**

CONSIGNE À L'INTERVIEWEUR : Lire les choix ou aider le répondant en lui indiquant de tenir compte de tous les membres du ménage.

1 Couple (avec ou sans enfants à la maison)
2 Famille monoparentale (avec enfant(s) de tout âge)
3 Autres (personnes non apparentées, colocataire, familles multiples, etc.)
98 Ne sait pas
99 Ne répond pas

FIN FILTRE D.

TC8A **Au cours des 12 derniers mois, quelle était votre occupation principale ?**

CONSIGNE À L'INTERVIEWEUR : Si nécessaire, lire les choix de réponses.

0 Travailleur(e) autonome → *FILTRE E : ALLEZ À LA TC8B*
1 Travailleur(e) à temps plein → *FILTRE E*

2 Travailleur(e) à temps partiel → *FILTRE E*
3 Étudiant(e)
4 Retraité(e) [rentier(ère)]
5 Semi-retraité(e) → *FILTRE E*
6 Personne tenant maison
7 En congé de maternité/paternité
8 Prestataire d'assurance-emploi
9 Prestataire d'aide sociale (sécurité du revenu)
10 Congé maladie / CNESST
11 Invalidité / SAAQ
12 Travailleur(e) saisonnier(ère)
13 Autre
98 Ne sait pas
99 Ne répond pas

TC8AA **Si réponse (97) à la TC8A, veuillez préciser : ____________________________.**

FILTRE E : SI RÉPONSES TRAVAILLEUR AUTONOME (00), TEMPS PLEIN (01), TEMPS PARTIEL (02) OU SEMI-RETRAITÉ (13) À LA TC8, ALLEZ À LA TC8B, SINON POURSUIVEZ À LA PROCHAINE SECTION.

TC8AAA **Avez-vous eu un emploi rémunéré dans les deux dernières années ?**
1 Oui → ALLER À TC8B
2 Non → ALLER à la prochaine section
9 Ne répond pas

TC8B **Travaillez-vous ou avez-vous travaillé dans le secteur de la santé et des services sociaux au cours des deux dernières années ?**
1 Oui → ALLER À TC8B1
2 Non
9 Ne répond pas

FIN FILTRE E.

TC8B1 **Travaillez-vous ou avez-vous travaillé en contact direct avec les usagers ?**
1 Oui
2 Non
9 Ne répond pas

TC8B4 **Combien de temps avez-vous cessé votre emploi en raison du confinement?**

1 Je n’ai pas cessé en raison du confinement
2 Moins d’une semaine
3 Une à deux semaines
4 Trois à quatre semaines
5 Un à trois mois
6 Trois à six mois
7 Plus de six mois
8 Je n’ai pas repris mon emploi
98 Ne sait pas
99 Ne répond pas

TC8B5 **Combien de temps avez-vous cessé votre emploi en raison de votre épisode de COVID-19 ?**

1 Je n’ai pas cessé en raison de mon épisode de COVID outre 2 semaines de confinement
2 Trois à quatre semaines
3 Un à trois mois
4 Trois à six mois
5 Plus de six mois
6 Je n’ai pas repris mon emploi
98 Ne sait pas
99 Ne répond pas

TC8B6 **Est-ce que vous avez dû changer d’emploi ou modifier votre emploi suite à votre épisode de COVID-19 ?**

1 J’occupe le même emploi selon un horaire plus demandant
2 J’occupe le même emploi selon les mêmes conditions
3 J’occupe le même emploi selon un horaire moins demandant
4 J’ai changé mon emploi pour un emploi plus demandant
5 J’ai changé mon emploi pour un emploi avec la même charge de travail
6 J’ai changé d’emploi pour un emploi moins demandant
98 Ne sait pas
99 Ne répond pas

BLOC 2 – ÉPISODE DE COVID-19

LA PROCHAINE SECTION CONCERNE VOTRE ÉPISODE DE COVID-19

COVID1 **Quel a été le degré de sévérité de votre épisode de COVID-19 ?**

**Léger**	1 Asymptomatique
	2 Symptomatique
**Modéré**	3 Patient hospitalisé sans oxygène
	4 Patient hospitalisé avec oxygène
**Sévère**	5 Patient hospitalisé aux soins intensifs
	6 Patient hospitalisé sous ventilateur (intubé)
	98 Ne sait pas
	99 Ne répond pas

TC11A_Acces **Avez-vous un médecin de famille ou une super-infirmière ?**

0 Oui, j'ai une super-infirmière
1 Oui, j'ai un médecin de famille
2 Oui, j'ai un médecin de famille et une super-infirmière
3 Non
8 Ne sait pas
9 Ne répond pas

COVID2 **Depuis votre épisode de COVID-19, combien de rendez-vous en externe avez-vous eu en lien avec des symptômes potentiellement reliés à la COVID-19 avec chacun de ces professionnels ?**

A Médecin
B Infirmier/ère
C Physiothérapeute
D Ergothérapeute
E Psychologue ou autre professionnel de la santé mentale
(Choix, 0, 1, 2, 3, 4, 5+) → ALLER À COVID2A si COVID2≥1

COVID2A **Combien de temps avec-vous attendu avant de voir ce professionnel entre votre épisode de COVID-19 et le premier rendez-vous ?**

A Médecin
B Infirmier/ère
C Physiothérapeute
D Ergothérapeute
E Psychologue ou autre professionnel de la santé mentale
Choix :

0) Pas de rendez-vous avec ce type de professionnel
1) Moins d'une semaine
2) Une à quatre semaines
3) Un à trois mois
4) Trois à six mois
5) Plus de six mois

COVID3 **Avec quels types de professionnels vous pensez avoir besoin d'un suivi pour des symptômes en lien avec votre épisode de COVID et pour lequel vous n'avez pas encore réussi à en obtenir un ?**

A Médecin
B Infirmier/ère
C Physiothérapeute
D Ergothérapeute
E Psychologue
(plusieurs choix possibles)

COVID4 **Parmi les éléments suivants, lesquels sont des difficultés que vous avez éprouvées pour obtenir des soins ?**

Répondez oui ou non pour chacune des difficultés suivantes.

	Oui (01)	Non (02)	Ne s'applique pas (07)	Ne répond pas (09)
**Difficultés avec le système de prise de rendez-vous ?**				
**Professionnels non disponibles (pas de plage) ?**				
**Problème de transport ?**				

COVID5 **Est-ce que le fait d'avoir eu un épisode de COVID-19 a changé votre opinion par rapport au respect des mesures sanitaires ?**

1 Plus respectueux des mesures sanitaires
2 Inchangé
3 Moins respectueux des mesures sanitaires
8 Ne sait pas
9 Ne réponds pas

COVID6 **Est-ce que le fait d'avoir eu un épisode de COVID-19 a changé votre opinion par rapport au vaccin ?**

1 Plus enclin à recevoir le vaccin
2 Inchangé
3 Moins enclin à recevoir le vaccin
8 Ne sait pas
9 Ne réponds pas

COVID7 **Selon vous, les vaccins COVID-19 sont-ils efficaces ?**

1 Pas du tout
2 Un peu
3 Assez
4 Très
8 Ne sait pas
9 Ne réponds pas

COVID8 **Selon vous, les vaccins COVID-19 sont-ils sécuritaires ?**

1 Pas du tout
2 Un peu
3 Assez
4 Très
8 Ne sait pas
9 Ne réponds pas

COVID9 **Selon vous, avez-vous reçu suffisamment d'informations vis-à-vis de ces vaccins pour décider de vous faire vacciner ou non (choix éclairé) ?**

1 Pas du tout
2 Un peu
3 Assez
4 Très
8 Ne sait pas
9 Ne réponds pas

COVID10 **Avez-vous été vacciné contre la COVID-19 ?**

0 Non
1 1 dose avant votre épisode de COVID-19
2 2 doses avant votre épisode de COVID-19
3 1 dose après votre épisode de COVID-19
4 2 doses après votre épisode de COVID-19
8 Ne sait pas
9 Ne réponds pas

**ACC _COVID1**	**En excluant le premier mois suite à votre diagnostic de COVID, avez-vous eu besoin de vous rendre à l'hôpital de manière urgente (imprévue) ?**<br>1 Oui → ALLEZ À LA QUESTION ACC_COVID2<br>2 Non → ALLEZ À SY_COVID1<br>98 Ne sait pas<br>99 Ne répond pas
**ACC _COVID2**	**De quel type de visite s'agissait-il ?**<br>1 Hospitalisation<br>2 Urgence<br>98 Ne sait pas<br>99 Ne répond pas

Parmi les symptômes suivants, lesquels avez-vous ressentis lors de votre diagnostic initial de COVID-19?

**SY_COVID1**	**Confusion ?**	1 Oui<br>2 Non<br>98 Ne sait pas<br>99 Ne répond pas
**SY_COVID2**	**Convulsions ?**	
**SY_COVID3**	**Fièvre ?**	
**SY_COVID4**	**Trouble de la parole ?**	

Parmi les symptômes suivants, lesquels avez-vous ressentis lors de votre épisode initial de COVID-19 et lesquels éprouvez-vous toujours à ce jour?

**SY_COVID5**	**Douleur aux articulations ?**	0 Non<br>1 Oui, disparu<br>2 Oui, toujours pareil<br>3 Oui, augmenté<br>4 Oui, diminué<br>5 Apparu plus d'un mois après l'épisode de COVID-19<br>98 Ne sait pas<br>99 Ne répond pas
**SY_COVID6**	**Maux de ventre ?**	
**SY_COVID7**	**Douleurs à la poitrine ?**	
**SY_COVID8**	**Mal d'oreilles ?**	
**SY_COVID9**	**Maux de gorge ?**	
**SY_COVID10**	**Toux ?**	
**SY_COVID11**	**Cracher du sang ?**	
**SY_COVID12**	**Écoulements nasaux ou nez bouché?**	

**SY_COVID13**	**Respiration bruyante ?**	
**SY_COVID14**	**Étourdissements ?**	
**SY_COVID15**	**Faiblesse ou engourdissement des jambes ou des bras ?**	
**SY_COVID16**	**Enflure des jambes ?**	
**SY_COVID17**	**Perte de l'odorat ?**	0 Non<br>1 Oui, disparu
**SY_COVID18**	**Perte du goût ?**	2 Oui, toujours pareil<br>3 Oui, augmenté
**SY_COVID19**	**Altération du goût ?**	4 Oui, diminué<br>5 Apparu plus d'un mois après
**SY_COVID20**	**Perte d'appétit ?**	l'épisode de COVID-19<br>98 Ne sait pas
**SY_COVID21**	**Nausées / vomissements ?**	99 Ne répond pas
**SY_COVID22**	**Difficultés à avaler liquides ou aliments ?**	
**SY_COVID23**	**Diarrhée ?**	
**SY_COVID24**	**Infection aux yeux ?**	
**SY_COVID25**	**Rash ou éruption cutanée ?**	

Nous allons maintenant vous poser quelques questions concernant votre fonctionnement dans vos activités de la vie quotidienne.

**FCT _COVID1**	**Quel énoncé décrit le mieux votre mobilité ?**<br>1 Je n'ai aucun problème à marcher<br>2 J'ai de légers problèmes à marcher<br>3 J'ai des problèmes modérés à marcher<br>4 J'ai de graves problèmes à marcher<br>5 Je ne peux pas marcher<br>98 Ne sait pas<br>99 Ne répond pas

FCT _COVID2 **Quel énoncé décrit le mieux votre habileté à compléter vos soins personnels ?**

1 Je n'ai aucun problème à me laver et m'habiller
2 J'ai de légers problèmes à me laver et m'habiller
3 J'ai des problèmes modérés à me laver et m'habiller
4 J'ai de graves problèmes à me laver et m'habiller
5 Je ne peux pas à me laver et m'habiller
98 Ne sait pas
99 Ne répond pas

FCT _COVID3 **Quel énoncé décrit le mieux votre habileté à exécuter vos activités habituelles ?**

1 Je n'ai aucun problème à faire mes activités habituelles
2 J'ai de légers problèmes à faire mes activités habituelles
3 J'ai des problèmes modérés à faire mes activités habituelles
4 J'ai de graves problèmes à faire mes activités habituelles
5 Je ne peux pas faire mes activités habituelles
98 Ne sait pas
99 Ne répond pas

FCT _COVID4 **Quel énoncé décrit le mieux votre douleur et inconfort ?**

1 Je n'ai ni de douleur ni d'inconfort
2 J'ai une douleur ou un inconfort léger
3 J'ai une douleur ou un inconfort modéré
4 J'ai une douleur ou un inconfort intense
5 J'ai une douleur ou un inconfort extrême
98 Ne sait pas
99 Ne répond pas

FCT _COVID5 **Quel énoncé décrit le mieux votre anxiété et dépression ?**

1 Je ne suis pas anxieux ou déprimé
2 Je suis légèrement anxieux ou déprimé
3 Je suis modérément anxieux ou déprimé
4 Je suis gravement anxieux ou déprimé
5 Je suis extrêmement anxieux ou déprimé
98 Ne sait pas
99 Ne répond pas

FCT _COVID6 **Quel énoncé décrit le mieux votre essoufflement ?**

1Je suis essoufflé seulement avec un exercice intense
2 Je suis à bout de souffle en me dépêchant ou en montant une légère pente
3 Je suis plus lent que la plupart des gens du même âge sur un terrain plat

4 Je m'arrête pour respirer en marchant 100m ou quelques minutes sur un terrain plat
5 Je suis trop essoufflé pour quitter la maison
98 Ne sait pas
99 Ne répond pas

Les prochaines questions se rapportent à votre fatigue. Les prochains énoncés seront évalués sur une échelle variant de totalement en désaccord à totalement en accord.

Durant la semaine passée, j'ai trouvé que…

FSS1 **Je suis moins motivé(e) quand je suis fatigué(e)**
1 Complètement en désaccord
2 En désaccord
3 Plutôt en désaccord
4 Neutre
5 Plutôt en accord
6 En accord
7 Complètement en accord
8 Ne sait pas
9 Ne répond pas

FSS2 **L'exercice physique me rend fatigué**
1 Complètement en désaccord
2 En désaccord
3 Plutôt en désaccord
4 Neutre
5 Plutôt en accord
6 En accord
7 Complètement en accord
8 Ne sait pas
9 Ne répond pas

FSS3 **Je suis facilement fatigué(e)**
1 Complètement en désaccord
2 En désaccord
3 Plutôt en désaccord
4 Neutre
5 Plutôt en accord

6 En accord
7 Complètement en accord
8 Ne sait pas
9 Ne répond pas

FSS4 **La fatigue gène mon fonctionnement physique**
1 Complètement en désaccord
2 En désaccord
3 Plutôt en désaccord
4 Neutre
5 Plutôt en accord
6 En accord
7 Complètement en accord
8 Ne sait pas
9 Ne répond pas

FSS5 **La fatigue me cause fréquemment des problèmes**
1 Complètement en désaccord
2 En désaccord
3 Plutôt en désaccord
4 Neutre
5 Plutôt en accord
6 En accord
7 Complètement en accord
8 Ne sait pas
9 Ne répond pas

FSS6 **Ma fatigue m’empêche d’avoir une activité physique soutenue**
1 Complètement en désaccord
2 En désaccord
3 Plutôt en désaccord
4 Neutre
5 Plutôt en accord
6 En accord
7 Complètement en accord
8 Ne sait pas
9 Ne répond pas

FSS7 **La fatigue m’empêche d’accomplir certains devoirs et responsabilités**
1 Complètement en désaccord

2 En désaccord
3 Plutôt en désaccord
4 Neutre
5 Plutôt en accord
6 En accord
7 Complètement en accord
8 Ne sait pas
9 Ne répond pas

FSS8 **La fatigue est parmi mes trois symptômes les plus invalidants**
1 Complètement en désaccord
2 En désaccord
3 Plutôt en désaccord
4 Neutre
5 Plutôt en accord
6 En accord
7 Complètement en accord
8 Ne sait pas
9 Ne répond pas

FSS9 **La fatigue interfère avec ma vie professionnelle et/ou familiale et/ou sociale**
1 Complètement en désaccord
2 En désaccord
3 Plutôt en désaccord
4 Neutre
5 Plutôt en accord
6 En accord
7 Complètement en accord
8 Ne sait pas
9 Ne répond pas

Veuillez répondre aux prochaines questions en considérant les dernières semaines. Les prochains énoncés seront évalués sur une échelle variant de peu ou pas à la majorité du temps.

SOFA1 **Je me sens fatigué pour une longue période après de l'activité physique**
1 Pas ou peu
2 Parfois
3 Bonne partie du temps
4 Majorité du temps
8 Ne sait pas
9 Ne répond pas

SOFA2 **Ma concentration est faible**

1 Pas ou peu
2 Parfois
3 Bonne partie du temps
4 Majorité du temps
8 Ne sait pas
9 Ne répond pas

SOFA3 **Mes muscles sont très fatigués après l'activité physique**

1 Pas ou peu
2 Parfois
3 Bonne partie du temps
4 Majorité du temps
8 Ne sait pas
9 Ne répond pas

SOFA4 **J'ai des maux de tête**

1 Pas ou peu
2 Parfois
3 Bonne partie du temps
4 Majorité du temps
8 Ne sait pas
9 Ne répond pas

SOFA5 **Je dois dormir pendant de longues périodes**

1 Pas ou peu
2 Parfois
3 Bonne partie du temps
4 Majorité du temps
8 Ne sait pas
9 Ne répond pas

SOFA6 **J'ai des douleurs musculaires après l'activité physique**

1 Pas ou peu
2 Parfois
3 Bonne partie du temps
4 Majorité du temps
8 Ne sait pas
9 Ne répond pas

SOFA7 **Je dors mal**

1 Pas ou peu
2 Parfois
3 Bonne partie du temps
4 Majorité du temps
8 Ne sait pas
9 Ne répond pas

SOFA8 **J'ai des troubles du langage (je me sens perdu dans mes mots)**

1 Pas ou peu
2 Parfois
3 Bonne partie du temps
4 Majorité du temps
8 Ne sait pas
9 Ne répond pas

SOFA9 **Ma mémoire est mauvaise**

1 Pas ou peu
2 Parfois
3 Bonne partie du temps
4 Majorité du temps
8 Ne sait pas
9 Ne répond pas

SOFA10 **J'ai des douleurs musculaires, même au repos**

1 Pas ou peu
2 Parfois
3 Bonne partie du temps
4 Majorité du temps
8 Ne sait pas
9 Ne répond pas

BLOC 3 – PROBLÈME DE SANTÉ LONGUE DURÉE

LES QUESTIONS QUI SUIVENT PORTENT SUR CERTAINS PROBLÈMES DE SANTÉ DE LONGUES DURÉES QUE VOUS POUVEZ AVOIR

Par « problème de santé de longue durée », on entend un état qui dure ou qui devrait durer 6 mois ou plus et qui a été diagnostiqué par un professionnel de la santé.

Consignes à l'interviewer : si la réponse est oui, interroger si la condition est pire, mieux ou identique depuis l'épisode de COVID-19 ou s'il s'agit d'un nouveau diagnostic.
Souffrez-vous de […]

		Non (01)	Oui (02)	Pire depuis COVID (03)	Mieux depuis COVID (04)	Idem depuis COVID (05)	Dx nouveau (06)
TC18A _SantéLD	Cancer (incluant les mélanomes et excluant les autres types de cancer de la peau) et ce, dans les 5 dernières années ?						
TC18B _SantéLD	Problèmes pulmonaires (asthme, bronchite chronique, emphysème, maladie pulmonaire obstructive chronique (MPOC)) ?						
TC18C _SantéLD	AVC (accident vasculaire cérébral), même mineur?						
TC18D _SantéLD	Maladie cardiaque (angine, infarctus, fibrillation auriculaire) ?						
TC18E _SantéLD	Insuffisance cardiaque ?						

TC18F _SantéLD	**Maladie de l'intestin (maladie de Crohn, colite ulcéreuse, diverticulite) ?**
TC18G _SantéLD	**Problèmes de foie ?**
TC18H _SantéLD	**Insuffisance rénale ?**

Rappelez-vous, nous sommes intéressés aux problèmes de santé diagnostiqués par un professionnel de la santé, notamment si vous prenez des médications pour ces conditions.

Souffrez-vous de […]

		Oui (01)	Non (02)	Pire depuis COVID (03)	Mieux depuis COVID (04)	Idem depuis COVID (05)	Dx nouveau (06)
TC18I _SantéLD	Problèmes musculosquelettiques vous causant douleur ou limitations ?						
TC18J _SantéLD	Arthrite ou polyarthrite rhumatoïde ?						
TC18K _SantéLD	Diabète ?						
TC18L _SantéLD	Hypertension ?						
TC18N _SantéLD	Trouble de l'humeur, tel que la dépression et le trouble bipolaire ?						
TC18O _SantéLD	Trouble d'anxiété ?						

TC18P _SantéLD	**Calcul automatique du score des maladies de longues durées.**
	EXPLICATION À L'INTERVIEWEUR : Un score des maladies de longues durées de 0 est indicatif d'une meilleure santé qu'un score de 15, lequel correspond à une réponse positive à tous les problèmes cités.

BLOC 4 – HABITUDES DE VIE

POURSUIVONS AVEC DES QUESTIONS SUR LES HABITUDES DE VIE

TC21 _FetL **Combien de fruits, de légumes ou de portions de ces produits mangez-vous chaque jour ?**

1 Cinq ou plus

2 Quatre

3 Trois

4 Deux

5 Moins de deux

8 Ne sait pas

9 Ne répond pas

TC22 _Habitudes **Avez-vous fumé une centaine de cigarettes (tabac) ou plus durant votre vie (environ 4 paquets) ?**

1 Oui

2 Non

98 Ne sait pas

99 Ne répond pas

TC23 _Habitudes **Actuellement, à quelle fréquence fumez-vous du tabac (incluant la cigarette, le cigare, le cigarillo et la pipe) ?**

1 À tous les jours

2 À l'occasion

3 Jamais

98 Ne sait pas

99 Ne répond pas

TC16A _FetL **Combien mesurez-vous sans chaussures ?**

Taille, valeur en pouces : ________________.

Si ne sait pas, ou ne répond pas, indiquez « NA ». Valeur comprise entre 41 et 89 pouces.

TC16B _FetL **Combien pesez-vous ?**

Poids, valeur en livres : ________________.

Si ne sait pas, ou ne répond pas, indiquez « NA ». Valeur comprise entre 75 et 550 livres.

TC16C _FetL ***Calcul automatique de l'IMC.***

BLOC 5 – CONSOMMATION D’ALCOOL ET DROGUES

LES PROCHAINES QUESTIONS PORTENT SUR LA CONSOMMATION D’ALCOOL ET DE DROGUES

TC22_Consom **Depuis votre épisode de COVID-19, combien de fois avez-vous bu 5 verres ou plus d’alcool à une même occasion ?**

1 Jamais
2 Moins d’une fois par mois
3 Une fois par mois
4 2 à 3 fois par mois
5 Une fois par semaine
6 Plus d’une fois par semaine
8 Ne sait pas
9 Ne répond pas

TC22A_Consom **Depuis votre épisode de COVID-19, comment a évolué votre consommation d’alcool ?**

1 J’ai arrêté d’en consommer
2 J’en consomme beaucoup moins souvent
3 J’en consomme un peu moins souvent
4 J’en consomme aussi souvent
5 J’en consomme un peu plus souvent
6 J’en consomme beaucoup plus souvent
8 Ne sait pas
9 Ne répond pas

TC23_Consom **Depuis votre épisode de COVID-19, à quelle fréquence avez-vous consommé du cannabis (marijuana, haschich, huile de haschich ou autres produits dérivés) à des fins récréatives ?**

1 Jamais
2 Une ou deux fois
3 3 à 11 fois par année
4 1 à 3 fois par mois
5 Une fois par semaine
6 Plus d’une fois par semaine
8 Ne sait pas
9 Ne répond pas

TC23A_Consom **Depuis votre épisode de COVID-19, comment a évolué votre consommation de cannabis ?**

1 J’ai arrêté d’en consommer
2 J’en consomme beaucoup moins souvent

3 J’en consomme un peu moins souvent

4 J’en consomme aussi souvent

5 J’en consomme un peu plus souvent

6 J’en consomme beaucoup plus souvent

8 Ne sait pas

9 Ne répond pas

BLOC 6 – CAPACITÉS CARDIOVASCULAIRES

LES PROCHAINES QUESTIONS PORTENT SUR L'ACTIVITÉ PHYSIQUE

DASI1 **Êtes-vous capable de monter les escaliers d'un étage à l'autre ou de monter une colline sans vous arrêter ?**

1 Oui
2 Non
8 Ne sait pas
9 Ne répond pas

DASI2 **Pouvez-vous encore faire des tâches plus difficiles comme laver le plancher, soulever ou déplacer des meubles lourds ?**

1 Oui
2 Non
8 Ne sait pas
9 Ne répond pas

DASI3 **Êtes-vous capable de faire des travaux dans la cour comme racler les feuilles, faire du sarclage, ou passer la tondeuse ?**

1 Oui
2 Non
8 Ne sait pas
9 Ne répond pas

DASI4 **Êtes-vous capable de participer dans des sports intenses comme la natation, le tennis en simple, le football, le basketball, le ski, le soccer, etc. ?**

1 Oui
2 Non
8 Ne sait pas
9 Ne répond pas

BLOC 7 – IMPACTS PSYCHOLOGIQUES DU CORONAVIRUS (COVID-19)

LES PROCHAINES QUESTIONS PORTENT SUR L'IMPACT PSYCHOLOGIQUE DU CORONAVIRUS

CONF1 **En général, comment trouvez-vous actuellement votre vie sociale, c'est-à-dire vos relations avec les gens qui vous entourent (parents, amis, connaissances)?**

1 Très satisfaisante
2 Plutôt satisfaisante
3 Plutôt insatisfaisante
4 Très insatisfaisante

Au cours du dernier mois, à quelle fréquence vous êtes-vous senti […]

EXPLICATION À L'INTERVIEWEUR : Lire les choix de réponses.

		Jamais (00)	Rarement (01)	Parfois (02)	La plupart du temps (03)	Tout le temps (04)
TC19A _VieQuo	Nerveux(se)					
TC19B _VieQuo	Désespéré(e)					
TC19C _VieQuo	Agité(e) ou ne tenant pas en place					
TC19D _VieQuo	Si déprimé(e) que plus rien ne pouvait vous faire sourire					
TC19E _VieQuo	Que tout était un effort					
TC19F _VieQuo	Être bon(ne) à rien					

Ne sait pas...98
Ne répond pas...99

TC19G _VieQuo ***Calcul automatique de l'indice de détresse psychologique.***

EXPLICATIONS À L'INTERVIEWEUR :
L'indice de détresse psychologique varie de 0 à 24. Plus l'indice est élevé, plus le niveau de détresse psychologique est élevé.

Rappelez-vous que toutes les informations que vous fournirez demeureront strictement confidentielles.

VC1 **En lien avec votre situation de couple, depuis votre épisode de COVID-19, la situation dans la relation conjugale est-elle…**

1 Beaucoup mieux
2 Un peu mieux
3 Restée la même
4 Un peu pire
5 Bien pire → ALLER À VC2
8 Ne sait pas
9 Ne répond pas

VC2 **Ressentez-vous le besoin que je vous transmette le contact de ressources pour parler à quelqu'un de cette situation ?**

Ressources d'aide :

L'Escale de l'Estrie
www.escaleestrie.com
819-569-3611 (Service 24/7)

La Méridienne (Estrie)
www.lameridienne.ca
1-888-699-3050

Centre féminin du Saguenay
418-549-4343

Carrefour pour femmes (Moncton)
506-875-5315

Centre de ressources et de crises familiales Beauséjour (Shediac)
506-533-9100

L'Accueil Sainte-Famille Inc. (Tracadie)
506-395-1500

BLOC 8 – ANXIÉTÉ ET STRATÉGIES DE GESTION

LA SECTION QUI SUIT PORTE SUR LES STRATÉGIES DE GESTION DE L'ANXIÉTÉ

STR1 **Si vous comparez votre degré d'anxiété d'aujourd'hui avec celui d'avant votre épisode de COVID-19, serait-il…**

1 Plus élevé
2 Moins élevé
3 Le même
8 Ne sait pas
9 Ne répond pas

STR2 **Que faites-vous pour diminuer votre anxiété et votre stress ?**

1 Activité physique
2 Activité culturelle
3 Consommation d'alcool
4 Drogue
5 Activité sociale
6 Consultation d'un professionnel de la santé mentale
7 Spiritualité
8 Relaxation
9 Autre
10 Aucun
98 Ne sait pas
99 Ne répond pas

BLOC 9 – PROCHES AIDANTS

LA SECTION QUI SUIT PORTE SUR LES PROCHES AIDANTS

PRA1A **Êtes-vous un proche aidant ?**

1 Oui → ALLEZ À PRA1B

2 Non → ALLEZ À PRA2A

8 Ne sait pas

9 Ne répond pas

PRA1B **Est-ce que l'épisode de COVID-19 vous a empêché de tenir votre rôle de proche aidant ?**

1 Oui, pendant la période d'isolement

2 Oui, je n'ai pas pu reprendre mon rôle à son niveau habituel

3 Non

8 Ne sait pas

9 Ne répond pas

PRA2A **Recevez-vous de l'aide d'un proche aidant ?**

1 Oui → ALLEZ À PRA2B

2 Non → ALLEZ AU PROCHAIN BLOC

8 Ne sait pas

9 Ne répond pas

PRA2B **La nécessité de cette aide est-elle reliée à votre épisode de COVID-19 ?**

1 Oui

2 Non

8 Ne sait pas

9 Ne répond pas

BLOC 10 – NOUVELLES

LES PROCHAINES QUESTIONS PORTENT SUR LES NOUVELLES ET LE CORONAVIRUS

NOUV1A **Sur une échelle de 1 à 10, trouvez-vous que les médias rapportent de manière adéquate ce qu'est l'expérience d'avoir la COVID-19 ?**

1 Désaccord complet
…
10 Accord complet
97 Je n'écoute pas les médias
98 Ne sait pas
99 Ne répond pas

Dans quelle mesure êtes-vous en accord avec les affirmations suivantes concernant la crise du coronavirus?

NOUV2A **Sur une échelle de 1 à 10, qvant mon épisode de COVID-19, j'estimais déjà posséder toutes les informations nécessaires pour me permettre de bien comprendre le coronavirus.**

1 Désaccord complet
…
10 Accord complet
97 Je n'écoute pas les médias
98 Ne sait pas
99 Ne répond pas

NOUV2B **Sur une échelle de 1 à 10, selon mon expérience, une fois que l'on a eu le coronavirus, on peut avoir des séquelles pour la vie.**

1 Désaccord complet
…
10 Accord complet
97 Je n'écoute pas les médias
98 Ne sait pas
99 Ne répond pas

BLOC 15 – GÉNÉRALITÉS 2

NOUS SOMMES RENDUS AU DERNIER BLOC DE QUESTIONS

TC4A **Êtes-vous né au Canada ?**

1 Oui

2 Non → FILTRE F : ALLEZ À LA QUESTION TC4B

8 Ne sait pas

9 Ne répond pas

FILTRE F : SI RÉPONSE NON (02) À LA TC4A, ALLEZ À LA TC4B, SINON POURSUIVEZ À LA GB4A.

TC4B **Depuis combien d'années vivez-vous au Canada ?**

1 4 ans et moins

2 5 ans et plus

8 Ne sait pas

9 Ne répond pas

FIN FILTRE F.

TC3B **Quel sexe vous a-t-on assigné à la naissance ?**

1 Masculin

2 Féminin

9 Ne répond pas

TC3B2 **Ce sexe correspond-il à votre genre (par genre, on entend le genre actuel, qui peut différer du sexe assigné à la naissance ou de celui inscrit dans les documents légaux) ?**

1 Oui

2 Non

3 Ne sait pas

9 Ne répond pas

OS1 **Quelle est votre orientation sexuelle ?**

1 Hétérosexuelle

2 Homosexuelle

3 Bisexuelle

4 Autre

8 Ne sait pas

9 Ne répond pas

TC9A **Quel est le plus haut niveau de scolarité que vous avez complété ?**

1 Aucun certificat, diplôme ou grade

2 Certificat d'études secondaires ou l'équivalent

3 Certificat ou diplôme d'apprenti ou d'une école de métiers

4 Certificat ou diplôme d'un collège, cégep ou autre établissement non universitaire

5 Certificat, diplôme ou grade universitaire

97 Autre

98 Ne sait pas

99 Ne répond pas

TC9AA **Si réponse autre (97) à la TC9A, précisez : ___________________.**

TC7 **Quel était approximativement le revenu total de votre ménage (incluant conjoint, conjointe) pour 2020 avant déduction d'impôt (ex : emploi, aide sociale, chômage, pension de retraite) ?**

1 Moins de 19 999$

2 20 000$ à 29 999$

3 30 000$ à 49 999$

4 50 000$ à 69 999$

5 70 000$ à 89 999$

6 90 000$ à 149 999$

7 Plus de 150 000$

8 Ne sait pas

9 Ne répond pas
